# Dynamic Second Harmonic Imaging of Proton Translocation Through Water Needles in Lipid Membranes

**DOI:** 10.1021/jacs.4c02810

**Published:** 2024-07-11

**Authors:** Seonwoo Lee, Chetan S. Poojari, Anna Maznichenko, David Roesel, Iwona Swiderska, Peter Pohl, Jochen S. Hub, Sylvie Roke

**Affiliations:** † Laboratory for fundamental BioPhotonics (LBP), Institute of Bioengineering (IBI), and Institute of Materials Science (IMX), School of Engineering (STI), and Lausanne Centre for Ultrafast Science (LACUS), École Polytechnique Fédérale de Lausanne (EPFL), Lausanne CH-1015, Switzerland; ‡ Theoretical Physics and Center for Biophysics, Saarland University, Saarbrücken 66123, Germany; § Institute of Biophysics, Johannes Kepler University Linz, Gruberstraße 40, Linz 4020, Austria

## Abstract

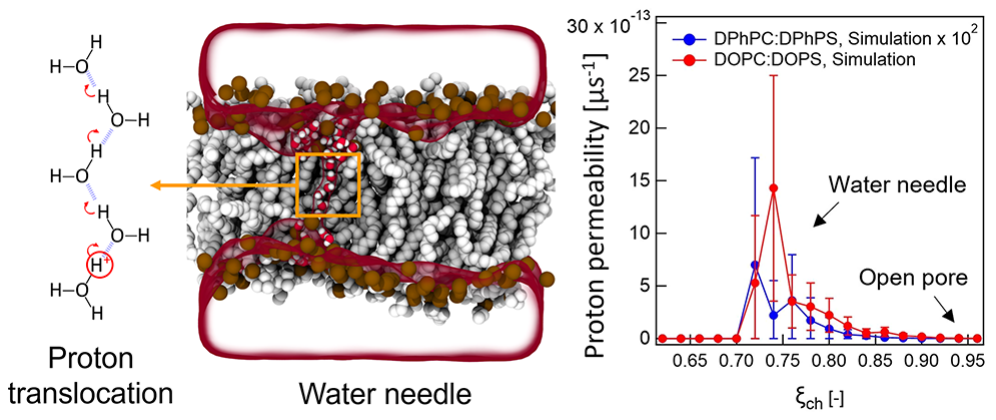

Proton translocation through lipid membranes is a fundamental process in the field of biology. Several theoretical models have been developed and presented over the years to explain the phenomenon, yet the exact mechanism is still not well understood. Here, we show that proton translocation is directly related to membrane potential fluctuations. Using high-throughput wide-field second harmonic (SH) microscopy, we report apparently universal transmembrane potential fluctuations in lipid membrane systems. Molecular simulations and free energy calculations suggest that H^+^ permeation proceeds predominantly across a thin, membrane-spanning water needle and that the transient transmembrane potential drives H^+^ ions across the water needle. This mechanism differs from the transport of other cations that require completely open pores for transport and follows naturally from the well-known Grotthuss mechanism for proton transport in bulk water. Furthermore, SH imaging and conductivity measurements reveal that the rate of proton transport depends on the structure of the hydrophobic core of bilayer membranes.

## 1 Introduction

**Figure 1:**
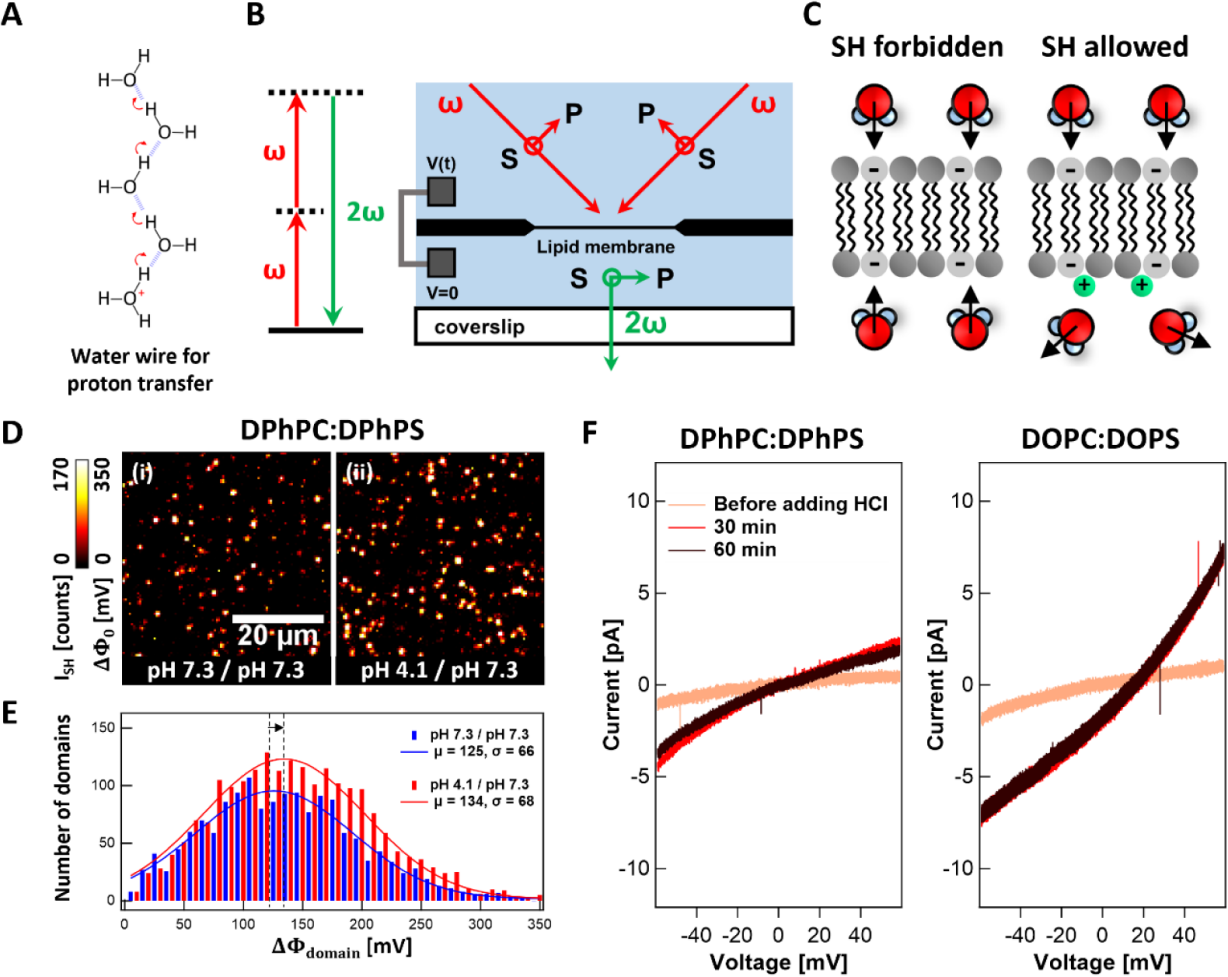
Membrane potential fluctuations and proton conductivity. (A) Illustration of proton transfer via the hydrogen network of water molecules. (B) Schematic illustration of the SH process and the optical geometry of the SH measurement. Two counterpropagating beams (1030 nm, 200 kHz, 190 fs, red arrows) are weakly focused on the lipid membrane at a 45° angle with respect to the surface normal. Phase-matched 515 nm SH photons (green) are emitted along the surface normal. (C) Schematic illustration of symmetric (forbidden coherent SH generation) and asymmetric (allowed coherent SH generation) hydration structure. The latter is induced by the interaction of H^+^ with charged PS headgroups on the bottom leaflet. (D) SH intensity and corresponding transmembrane potential difference (ΔΦ_0_) images (acquisition time: 1 s) of a symmetric membrane composed of a 70:30 mol % DPhPC:DPhPS mixture surrounded by the aqueous solution: (i) the top and bottom leaflets are in contact with a 50 mM KCl solution at a bulk pH of 7.3 each with 10 mM phosphate buffer solution and (ii) 10 min after the bottom compartment pH is varied to 4.1. (E) Domain analysis in terms of the occurrence of transmembrane potential difference values (detailed in Supporting Information S9), showing the number of domains observed in 20 × 1 s SH image frames at the pH environments of Figure 1D. Data points are fitted with Gaussian distribution, and the mean (μ) and standard deviation (σ) are displayed. The shift in the entire distribution is indicated by the arrow. (F) Electrical conductivity of 70:30 mol % DPhPC:DPhPS and 70:30 mol % DOPC:DOPS measured with the addition of (HCl)_aq_ to the bottom compartment, such that the pH = 4.1.

Proton (H^+^) transport is vital to the cellular energy production of plants and animals. In mitochondrial membranes, adenosine triphosphate (ATP) synthases use proton gradients to transport protons and produce ATP.^1,2^ For efficient ATP synthesis, the membrane should control the flux of protons so that it is concentrated on the synthases. The proton permeability of the lipid membrane, however, is known to be several orders higher in magnitude than that for any other cations, which potentially leads to a major energy loss, if it is not controlled.^3−5^ To understand the translocation of protons through lipid bilayer membranes (LBMs), numerous studies have been performed using fluorescence microscopy^6−10^ and conductivity measurements.^11−13^ These studies have proposed a number of models of possible membrane transport mechanisms,^14^ namely, the solubility-diffusion model,^5,10^ the weak-acid model,^15,16^ the fatty acid flip-flop model,^17,18^ and the thermal pore formation model.^5,19^ Although the exact mechanism of proton transport is still under investigation, the movement of a proton across membranes is likely influenced by how it is transported in bulk water. In pure bulk water, protons use the correlated hydrogen bond network of water to “hop” between water molecules, resulting in a fast and sequential proton transfer mechanism,^20−22^ known as the Grotthuss mechanism (Figure 1A). Due to this intrinsic structural and dynamical advantage, proton mobility in pure water is at least 5–7 times higher than that of other cations.^23,24^ Molecular dynamics (MD) simulations have shown that transient continuous chains of hydrogen bonds (so-called “water wires”) are formed in the phospholipid membrane.^25^ Water molecules within the wires act in a cooperative manner, aligning themselves in a preferential direction. Therefore, the higher proton permeability of membranes may be attributable to a transport mechanism involving water wires. The existence of water wires in membranes is, however, uncertain, as it requires the energy-consuming process of the opening and closing of transient structural defects.

Recently, we combined MD simulations with label-free high-throughput second harmonic (SH) imaging to study the transport of divalent cations through free-standing and giant unilamellar vesicle phospholipid membranes.^26,27^ Nonresonant SH generation, a process in which two photons with frequency ω are converted into a photon with frequency 2ω, illustrated in Figure 1B, has, by virtue of its spatial symmetry selection rule, a unique sensitivity to interfaces. Only anisotropic molecular arrangements of nonisotropic molecules generate coherent SH photons. Therefore, coherent SH photons are uniquely generated from an interface if it is sandwiched between isotropic media. Likewise, as Figure 1C illustrates, a symmetric bilayer generates no coherent SH photons while an asymmetric bilayer does. Although already exploited since the 1970s,^28^ SH imaging has become a major tool for probing structural polar assemblies of proteins for life science diagnostics.^29,30^ Interfacial studies have also been performed,^31−34^ but with less abundance because of the low number of photons that are typically generated from an interface. Exploiting resonance enhancement^35,36^ or structural harmonophores^37^ are ways to overcome this weakness. However, the presence of probes potentially modifies the molecular architecture of the system, which prevents one from examining its molecular properties. Recently, a wide-field SH imaging approach was developed, which increased the SH imaging throughput by a factor of >5000.^38,39^ This wide-field imaging approach made it possible to record nonresonant SH images with 400 nm spatial resolution and subsecond acquisition times. Because the interactions are nonresonant (illustrated in Figure 1B), no a priori specificity is obtained from aqueous systems. However, it turns out, as explained below, that because of this aspect two essential features stand out: (1) with this type of imaging, interfacial water is specifically detected and (2) the recorded intensity can be converted into transmembrane potential values. For a nonresonant nonlinear optical interaction, the molecular response is independent of the molecular species, and the number of nonzero nonlinear optical parameters is small, with each of these being frequency independent.^40^ Water outnumbers every other molecule at nearly every interface, with typical ratios of 100:1,^41,42^ giving rise to SH intensity contributions of 10^4^:1 in favor of water.^41,42^ High-throughput nonresonant SH imaging is therefore nearly always a probe of interfacial water, which has been exploited for solid–liquid, liquid–liquid, and membrane interfaces.^39,43−46^ Interfacial water orientation is determined by chemical interactions and electrostatic field interactions, and those interactions are separately included in nonlinear optical equations, allowing for the quantification of the interfacial water order due to chemical interactions (via the second-order surface susceptibility, ) as well as the surface potential (Φ_0_, via the effective third-order susceptibility χ^(3)^′, which is a known quantity for water).^39^ For free-standing lipid bilayer membranes in an aqueous solution, it was shown that transmembrane potential (ΔΦ_0_) images and videos can be constructed, with the average values agreeing very well with capacitance minimization.^47^

Both aspects, the imaging of water and the ability to create electrostatic transmembrane potential videos, allow for a unique insight into the biophysics of membranes. Using this method, in combination with capacitance minimization, membrane potential fluctuations on liquid lipid membranes were discovered. We found that these fluctuations occur in a variety of contexts, with different ions,^47^ types of symmetries,^48^ and different model membrane systems,^26,27^ and therefore seem to represent a universal aspect of charged phospholipid bilayer membranes. We explain the potential fluctuations in the context of concentrated confined electrolyte solutions: the hydrated charged headgroup volume that surrounds the hydrophobic core of the membrane essentially consists of a crowded electrolyte solution, which exhibits intrinsic nonstatistical distributions of ions.^49−54^ An important consequence of these fluctuations is that the degree of ionization of the membrane varies locally in space and time, which leads to transient intrinsic membrane potential gradients. Thus, an internally generated electrostatic field gradient exists temporarily across the membrane interface. Using MD simulations combined with free energy calculation techniques for pore formation,^55,56^ we showed that these gradients dramatically impact the energetics of pore formation, temporarily lowering the energy barrier for pore formation to the level needed to allow for the transport of divalent cations across certain membranes.^26,27^

Here, we investigate whether a similar mechanism plays a role in proton translocation. We start by characterizing two types of symmetric lipid membranes composed of DPhPC:DPhPS (1,2-diphytanoyl-*sn*-glycero-3-phosphocholine, 1,2-diphytanoyl-*sn*-glycero-3-phospho-l-serine) or DOPC:DOPS (1,2-dioleoyl-*sn*-glycero-3-phosphocholine, 1,2-dioleoyl-*sn*-glycero-3-phospho-l-serine) (see Supporting Information S1 and Figure S1 for their structures), in terms of their transient membrane potential landscape. Using conductivity measurements, we determine their permeability to protons with DOPC:DOPS being more permeable than DPhPC:DPhPS. SH imaging this H^+^ transport, we observe a concomitant change in the water structure during transport with DOPC:DOPS exhibiting more H^+^-induced changes than DPhPC:DPhPS. Based on these images, we extract translocation rates, which vary across the images and are different for DOPC:DOPS and DPhPC:DPhPS membranes. MD simulations show that proton translocation can occur via thin water needles and does not require completely open pores under the transmembrane potential fluctuations of the magnitude found in the SH imaging. Since the formation of water needles is far more likely than the formation of open pores according to free energy calculations,^57^ H^+^ permeation mostly proceeds along water needles. The comparison of proton permeabilities computed from the MD simulations and the experimentally deduced values reveals good qualitative agreement, suggesting that proton transport via the Grotthuss mechanism along the water needles is a highly plausible mechanism for proton transport across phospholipid membranes.

## 2 Results and Discussion

### 2.1 Transient Membrane Potential Fluctuations and Proton Conductivity

We investigate transient membrane potential fluctuations using free-standing lipid bilayer membranes (LBMs) formed via the Montal-Müller method.^58^ The modifications required to form horizontal instead of vertical membranes^59^ are described in more detail in the Supporting Information S2. The lipid membranes are SH imaged with a medium repetition rate, wide-field nonlinear SH microscope. The optical layout, illustrated in Figure 1B, involves two counterpropagating beams, each incident under 45° with respect to the surface normal of the lipid membrane (details can be found in Supporting Information S3). Symmetric lipid membranes composed of 70:30 mol % DPhPC:DPhPS and 70:30 mol % DOPC:DOPS were formed with the top/bottom leaflet in contact with a 50 mM KCl solution having an identical pH for both leaflets (pH = 7.3/pH = 7.3) with a 10 mM sodium phosphate buffer (Na_2_HPO_4_–NaH_2_PO_4_) or having a different pH value for both leaflets (pH = 4.1/pH = 7.3). Figure 1D shows single-frame SH images (*I*_SH_) with the corresponding transmembrane potential differences (ΔΦ_0_) also indicated in both situations. Note that image (ii) was acquired 10 min after changing the pH of the bottom compartment by adding (HCl)_aq_. The acquisition time for individual frames was 1 s. The data for DOPC:DOPS are shown in Figure S3. Details about the conversion of SH intensity to the membrane potential can be found in Supporting Information S6. Both images in Figure 1D show SH contrast, even though (i) is on average symmetric around the surface plane. The reason for this observation is that local spatial symmetry is broken, induced by spatially and temporally varying charge distributions in the top and bottom leaflets. Increasing the acquisition time reduces the effect of such spatiotemporal variations, resulting in a vanishing of SH intensity (as shown in Figure S5). Introducing a pH gradient by adding (HCl)_aq_ to the bottom compartment, such that the total ionic strength changes to 61 mM, leads to an increase in the SH intensity. Note that this is not caused by the small difference in ionic strength (Supporting Information S8, Figure S6). The increase is likely caused by the protonation of PS headgroups, which have an average p*K*_a_ value of 5.5.^60^ This alters the charge on the membrane and also the orientation of interfacial water in the bottom compartment, thereby increasing the SH intensity. Movie S1 shows a time-lapse of *I*_SH_/ΔΦ_0_ corresponding to Figure 1D. As observed previously,^26,27,48^ also here, the intensity fluctuations are uncorrelated in time, indicating that they originate over time scales shorter than the acquisition time of 1 s.

To obtain more insight into the transmembrane potential fluctuations, we performed a single-domain analysis. Using 20 consecutive frames, domains are chosen by taking into account their intensity and their individual sizes. Figure 1E shows the number of domains observed in SH images before and after the addition of (HCl)_aq_ for the DPhPC:DPhPS case as a function of the potential difference. We find transmembrane potential values reaching up to 350 mV. Note that this value is significantly higher than the average potential of 50 mV across the entire membrane, calculated considering the membrane area and the average SH intensity. The data for DOPC:DOPS membranes are shown in Figure S7 and show a similar influence of protons on the *I*_SH_/ΔΦ_0_ distributions. These locally high values of the surface potential result from the locally higher amounts of protonation of PS groups. A transmembrane potential difference of 350 mV in a 60 mM ionic strength solution is only possible if a condensed ion or Stern layer is present.^38^Figure S8 displays computations using the Gouy–Chapman-Stern (GCS) model that shows this aspect in more detail. Adding protons generates new SH domains and distorts the distribution to higher values for the DPhPC:DPhPS and DOPC:DOPS membranes, respectively (indicated by the arrow in Figures 1E and S7). It also shows that when protons are present at the interface, there is a larger spread in the transmembrane potential distribution with more occurrences of higher potential values.

Having shown that protons interact with both lipid membranes, we next determine whether and by how much they translocate through the membrane. We do this by performing conductivity measurements, as described in detail in Supporting Information S4. Starting with a membrane placed in between identical solutions, we again lower the bottom compartments’ pH to 4.1 by adding (HCl)_aq._ Before and after lowering the pH of the bottom compartment from pH 7.3 to pH 4.1, the membrane conductivity is measured at 30 min time intervals.

Figure 1F shows the resulting current–voltage (I–V) curves for DPhPC:DPhPS and DOPC:DOPS. The slope of the I–V curves is the conductivity, which increases after the pH drops. The reversal potential, the voltage at which there is no net flow of current across the membrane, increases upon the addition of (HCl)_aq_. It should be noted that the observed change in conductivity is not attributed to the small difference in ionic strength between the leaflets (Supporting Information S8, Figure S6). The increased conductivity thus suggests that the membrane conducts charges that presumably are Cl^–^ and H^+^ ions. The average ratio of H^+^ to Cl^–^ permeability (*P*_H_+/*P*_Cl_−) can be estimated from the reversal potential (*V*_m_) using the Goldman equation:
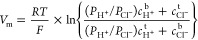
1where *R* is the ideal gas constant, *T* is the temperature, *F* is the Faraday’s constant, and and  are the ion concentrations of the bottom and top compartments, respectively. Upon the addition of (HCl)_aq_, DOPC:DOPS and DPhPC:DPhPS exhibit maximum reversal potentials of 15 and 2 mV, respectively, implying that both types of membrane conduct H^+^ more than 50 times better than Cl^–^ (see Supporting Information S11). These findings suggest that the average permeation rate of protons is significantly higher than that of chlorides, which can be explained by a higher activation energy for Cl^–^ permeations.^61^ Therefore, the increase in conductivity (Figure 1F) is primarily driven by the translocation of protons.

Next, we quantify the proton conductivity, permeability, and the number of permeating protons. Using the slope of the graph and membrane area (with *R* = 55 μm, 9503 μm^2^), we find that DOPC:DOPS has a higher conductivity (2.4 × 10^–7^ S/cm^–2^) than DPhPC:DPhPS (1.5 × 10^–7^ S/cm^–2^) at an identical pH for both leaflets (pH = 7.3). The values are in good agreement with the literature.^62^ Upon the addition of (HCl)_aq_, DOPC:DOPS exhibits a conductivity up to twice as high (11.2 × 10^–7^ S/cm^–2^) as DPhPC:DPhPS (5.5 × 10^–7^ S/cm^–2^) (see Supporting Information S12, Figure S9A).

The proton permeability () is computed using the Goldman–Hodgkin–Katz flux equation:^14^
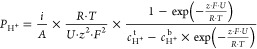
2where *i* is the current for a given external voltage *U*, *A* is the membrane area, and *z* is an ion of valency. Based on the aforementioned H^+^ to Cl^–^ permeability ratios and the I–V graphs with the maximum slopes, we obtain a proton-induced current of −7.6 pA for DOPC:DOPS and −4.6 pA for DPhPC:DPhPS under an external bias of −0.06 V. Using these values, we find a higher permeability for DOPC:DOPS (= 3.7 × 10^–6^ cm/s) than DPhPC:DPhPS (), showing a good agreement with the difference in the conductivity results. The obtained proton permeability also agrees with previously reported values (10^–8^–10^–6^ cm/s).^10,63−65^ It should be noted that the contribution of the unstirred layers (ULs) to the measured permeability is not significant because the proton permeability of the membrane is much lower than that of the unstirred liquid.^14^ With a diffusion coefficient of the proton carrier, i.e., buffer molecules, (*D*_p_ ∼5 × 10^–6^ cm^2^/s) and a typical unstirred layer thickness (δ ∼200 μm), we estimate the permeability in unstirred liquid layers, at .

In the absence of an externally applied potential, proton translocation can be driven by a proton concentration gradient. Figure 1F shows that the current at a zero external bias (*U* = 0 mV) increases upon the addition of (HCl)_aq_. Unilateral protonation of the PS headgroup increases the surface potential differences across the membrane, while the concentration gradient of protons and chlorides creates *V*_m_ (eq 1), which has the opposite sign to the surface potential.^14^ Proton permeation also affects *V*_m_ by changing the concentration gradient and modifies the surface potential by proton binding to PS molecules on the opposite side of the membrane. The number of transported protons per second (*n*) is given by *n* = *I*/*e*, where *I* is the current at *U* = 0 mV, and *e* is the elementary charge. Considering the change in current with time (Figure S9B), after 1 h, ∼5.7 × 10^–14^ moles of H^+^ have transferred for DOPC:DOPS while ∼0.7 × 10^–14^ moles of H^+^ for DPhPC:DPhPS. Furthermore, a negligible change in pH value is found after 1 h, in agreement with this result.

The observed difference in proton conductivity, permeability, and the number of permeated protons between both membranes suggests that proton translocation rates are influenced by the hydrophobic structure of the bilayers as the headgroup composition is identical. As shown in Figure S1, DPhPC:DPhPS phospholipids are fully saturated branched lipids. Their methyl groups can cause steric hindrance to proton translocation. In contrast, DOPC:DOPS has unsaturated alkyl chains and no methyl groups. A more open structure is allowed with a less attractive force between the chains, reducing the hydrophobic barrier for proton transfer. Our results are supported by proton permeation studies using fluorescence microscopy where a higher proton permeation has been found for nonbranched lipid membranes compared to branched lipid membranes.^9^ Qualitatively, this difference is also in agreement with a trend we observed earlier for divalent ions translocating through giant unilamellar vesicle membranes^26,27^ and can be explained by the difference in the free energy barrier that protons experience in traversing both hydrophobic cores. We will revisit this difference when we perform the simulations.

### 2.2 SH Imaging of Proton Translocation

**Figure 2:**
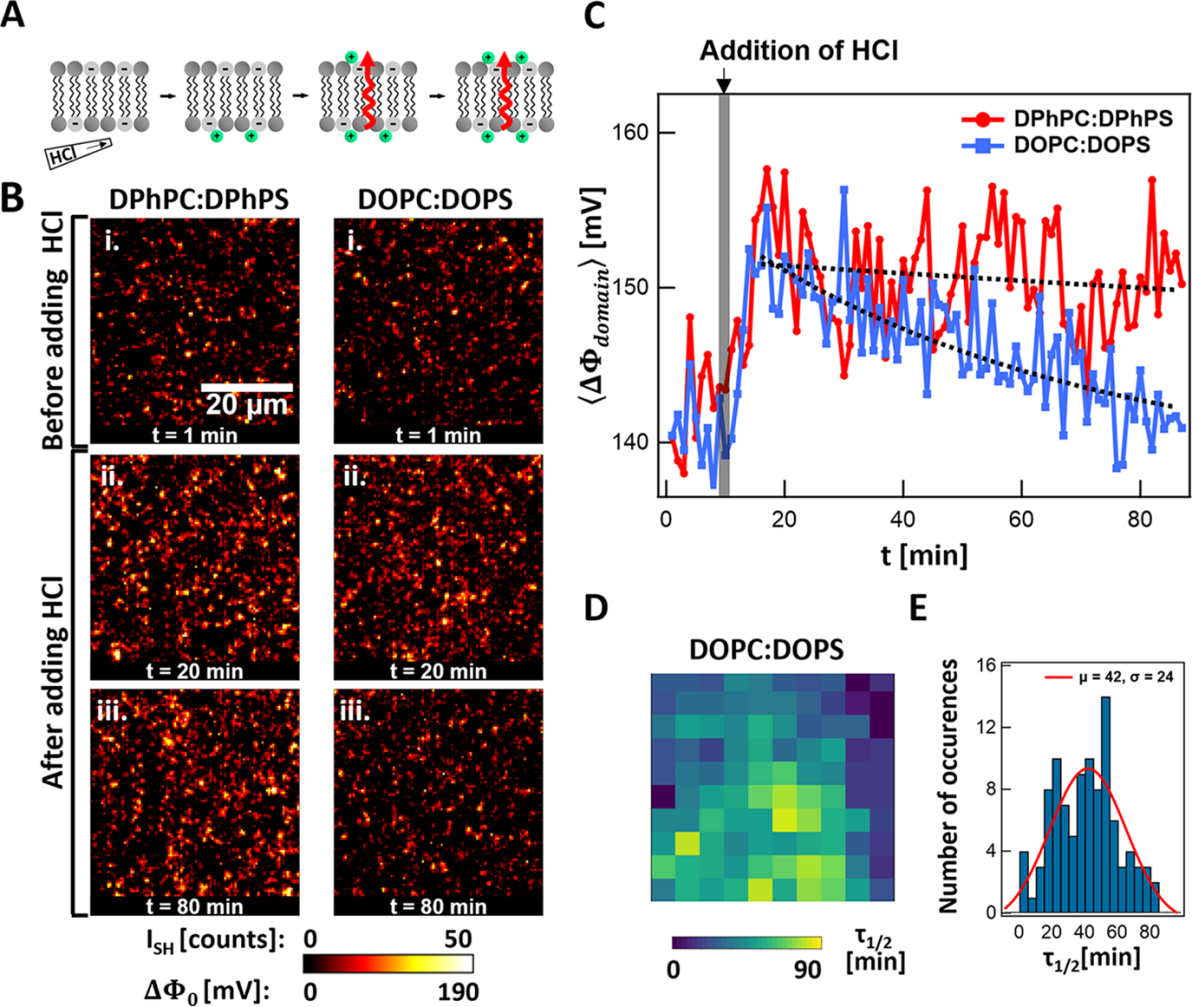
SH imaging of H^+^ translocation-induced membrane hydration changes. (A) Illustration of proton translocation through lipid membranes. (B) Time series of SH images of symmetric membranes composed of 70:30 mol % DPhPC:DPhPS (left) and 70:30 mol % DOPC:DOPS (right) before and after the addition of (HCl)_aq_ to the bottom compartment. Both sides of the bilayer are initially in contact with a pH 7.3, 50 mM KCl, and 10 mM phosphate buffer solution. Upon the addition of (HCl)_aq_, the pH value of the bottom compartment decreases from pH 7.3 to 4.1, while the pH value at the top compartment remains at pH 7.3. The SH images are obtained with 20 × 1 s frame averages, and all beams are P-polarized. The scale bar (20 μm) is the same for all images. (C) Average surface potential per domain (⟨ΔΦ_domain_⟩) observed over time for DPhPC:DPhPS and DOPC:DOPS with the addition of (HCl)_aq_ at *t* = 10 min. (D) The map of exponential decay time constants (τ_1/2_) for 100 different ROIs with a size of 4.5 μm × 4.5 μm on a DOPC:DOPS membrane. (E) Histogram of the time constants together with fitted Gaussian distributions, and the mean (μ) and standard deviation (σ) are displayed.

Next, we SH image the concomitant change in the water structure during proton translocation. To do so, we add (HCl)_aq_ to the bottom side of membranes, using the same concentration as in Figure 1, as illustrated in Figure 2A. Figure 2B shows SH images of 70:30 mol % DPhPC:DPhPS and DOPC:DOPS before (*t* = 1 min) and after (*t* = 20 and *t* = 80 min) addition of (HCl)_aq_. The SH images correspond to the average of 20 consecutive single 1 s frame images (1 s/frame). Note that the SH intensity of each domain in Figure 2B is on average lower than that of the single frame image in Figure 1D. This is the result of averaging the intensities of several domains that appeared in 20 s. Considering the SH intensity and size of domains, we next plotted the average transmembrane potential/domain ⟨ΔΦ_domain_⟩ as a function of time. Movies of the proton translocation-induced membrane hydration are included (Movie S1 for DPhPC:DPhPS and Movie S2 for DOPC:DOPS).

As Figures 2B and 2C show, when (HCl)_aq_ is added, the SH intensity for DOPC:DOPS and DPhPC:DPhPS increases. As explained above, the increase in intensity arises from the protonation of PS lipids on one of the leaflets, which changes the orientational distribution of water molecules on that leaflet. After reaching the maximum SH contrast, SH intensity gradually decreases. DOPC:DOPS exhibits a more significant drop in intensity compared to DPhPC:DPhPS. The decrease in SH intensity can only be achieved by restoration of the initial amount of water structural anisotropy and likely connects with proton permeation, since on the same time scale, we have seen that the conductivity changes (Figure 1F).

Once protons have reached the other leaflet, H^+^-PS binding also occurs on this side, restoring the difference in the water interfacial structure. The process is illustrated in Figure 2A. SH imaging provides information about how many protons will bind to the second leaflet after crossing the membrane. Assuming the membrane can be modeled as a parallel plate capacitor in contact with aqueous solutions, for each domain, the potential difference (ΔΦ_domain_) can be converted to the difference in surface charge density between the top and bottom leaflets (Δσ_domain_) where Δσ_domain_ = *C* ΔΦ_domain_, with *C* = ε_0_ε/*d*, ε is the dielectric constant (ε = 2.1) and *d* is the thickness of the hydrophobic core (*d* = 4 nm).^48^ Protons binding to the top leaflet would decrease the Δσ_domain_. From a decrease in Δσ_domain_, we find the number of protons bound to the top leaflet (*N*_domain_) changes where *N*_domain_ = (Δσ_domain_*A*_domain_)/*e*, where *A*_domain_ is the domain size and *e* is the elementary charge. Using both the potential difference and size of domains, after 1 h, ∼1.1 × 10^–17^ moles of charges have been neutralized on the top leaflet for DOPC:DOPS while ∼0.2 × 10^–17^ moles of H^+^ for DPhPC:DPhPS. Combined with the results of conductivity measurement shown in Figure 1F, this shows that only a small fraction of the translocated protons remain at the membrane interface: 1 out of 5.2 × 10^3^ for DOPC:DOPS and 1 out of 3.2 × 10^3^ for DPhPC:DPhPS, respectively.

The decay rate of SH intensity/transmembrane potential is correlated to the translocation rate of the protons. Fitting the decaying part of the curves in Figure 2C with exponential decays, we obtain decay constants (τ_1/2_) for DOPC:DOPS (=67 min) and DPhPC:DPhPS (=442 min). Considering the proportion of protons remaining at the interface upon permeation, the decay constants translate into fluxes (*F*_LBM_) of 7.5 × 10^6^ ions/s (DOPC:DOPS) and 0.8 × 10^6^ ions/s (DPhPC:DPhPS). The difference in fluxes indicates that nonbranched unsaturated lipids have a higher proton permeability compared to branched saturated lipids, which is in agreement with the conductivity measurements. Furthermore, the values of fluxes can be converted into proton permeability (), which is determined by:
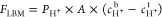
3where *A* is the membrane area. Based on eq 3, we extracted a higher proton permeability for DOPC:DOPS (1.7 × 10^–6^ cm/s) than that for DPhPC:DPhPS (0.2 × 10^–6^ cm/s). Proton permeability extracted from SH images and the current measurements are comparable.

The spatiotemporal proton movement across the membrane was examined for DOPC:DOPS by considering the local decay of SH intensities for 100 different 4.5 × 4.5 μm regions of interest (ROIs). The time constants of DOPC:DOPS were obtained by fitting the intensity decay of each ROI with an exponential curve. Figure 2D shows a spatial map of the obtained decay times. The corresponding histogram of the time constants is shown in Figure 2E, and the Gaussian fitting function reveals an average translocation time constant of 42 min and a standard deviation of 24 min. This corresponds to average ion fluxes of 1.2 × 10^7^ ions/s and a standard deviation of 1.0 × 10^7^ ions/s. Thus, translocation is a spatiotemporally inhomogeneous process.

### 2.3 MD Simulations of Proton Translocation across Water Needles

**Figure 3:**
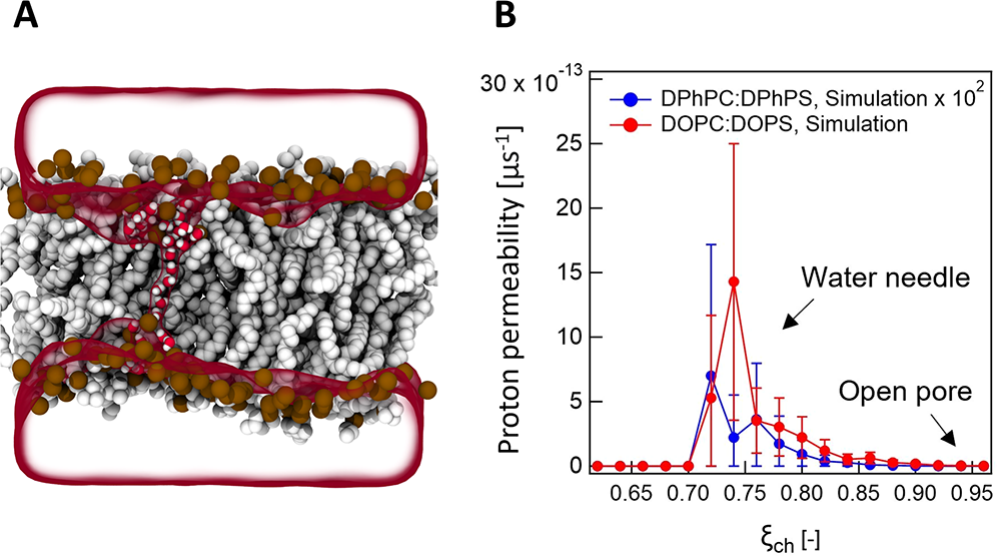
Proton translocation mechanism. (A) MD snapshot of an aqueous defect formation at a transmembrane potential of 300 mV in a DOPC:DOPS membrane. Phosphorus atoms of lipids are rendered as brown spheres, hydrophobic tails as gray spheres, and water molecules inside the membranes as red/white spheres, with bulk water as a transparent surface. (B) Computed proton permeability of DPhPC:DPhPS (blue) and DOPC:DOPS (red) as a function of chain coordinate ξ_ch_, where ξ_ch_ determines the connectivity of defects through the membrane.

We implemented atomistic molecular dynamics (MD) simulations to understand the mechanism of proton translocation. As noted above, based on the Grotthuss mechanism, we hypothesize that proton translocation can occur via transient water wires/needles. A snapshot of such a wire is shown in Figure 3A. The existence of water wires requires temporary defect formation within the membrane. To understand this process, we first computed the potential of mean forces (PMFs, also known as free energy profile) of defect formation across 70:30 mol % DOPC:DOPS and 70:30 mol % DPhPC:DPhPS membranes at transmembrane potentials between 0 and 300 mV, as these are the observed transmembrane potential values derived from the SH images (see Supporting Information S13 and S14 for more details on PMF calculations).

PMFs of defect formation as a function of chain coordinate ξ_ch_ are shown in Figure S10. ξ_ch_ is a coordinate that quantifies the degree of connectivity of defects across the bilayer for 0.1 < ξ_ch_ ≲ 0.8 or the size of the defect for ξ_ch_ ≳ 0.8. ξ_ch_ = 0.1 and ξ_ch_ = 1 correspond to an unperturbed membrane and to a completely open pore, respectively.^55,57^ PMF calculations along ξ_ch_ have previously been used to quantify the effects of membrane-active peptides^66^ or polymers,^67^ small molecules,^68^ or electric fields^69^ on pore formation. The MD snapshot of Figure 3A shows a transient aqueous defect (water needle) obtained at ξ_ch_ = 0.74. Figure S10 shows that the free energy for wire formation in DPhPC:DPhPS is significantly higher as compared to that for wire formation in DOPC:DOPS. Since the free energy Δ*G* translates to a probability via exp(−βΔ*G*), where β is the inverse temperature, this shows that defects are more likely to occur in nonbranched DOPC:DOPS while the probability of defect formation decreases in the presence of branched alkyl chains of DPhPC:DPhPS. These trends agree qualitatively with the data of Figures 1 and 2.

By computing PMFs of pore formation in the presence of transmembrane potentials, we observe that potentials up to 300 mV, as observed from our SH data, have only a small effect on the free energy of the water needle formation (Figure S10). Indeed, a simple analytic model for the energy of the water needle shows that needles with radii up to 3 Å are stabilized by a transmembrane potential of 300 mV by only ∼1 kJ/mol (Figure S11). This observation can be attributed to the fact that the stabilization of a defect increases quadratically with both the membrane potential and defect radius. Consequently, the moderate potential of 300 mV and the small size of defects exert only little impact on the formation of a water needle.

According to the PMFs, the formation of a wire across DPhPC:DPhPS or DOPC:DOPS (ξ_ch_ = 0.74) is more likely as compared to the formation of a completely open defect (ξ_ch_ > 0.95). To quantify the contributions of defects of different sizes to H^+^ flux, we performed a series of simulations restrained at various degrees of pore opening between ξ_ch_ = 0.62 and ξ_ch_ = 0.96 in steps of 0.02, and we extracted the flux of hydronium ions at a constant transmembrane potential of 300 mV. In this study, we simulated H^+^ flux using a classical hydronium model without taking the Grotthus mechanism into account. The calculated H^+^ flux may thus be lower compared to the experimental value, but the comparison of H^+^ flux among different pore sizes or different lipid compositions is a reasonable assumption. Figure S12 shows an increase in proton flux through DOPC:DOPS and DPhPC:DPhPS with increasing ξ_ch_. In the range of ξ_ch_ values between 0.72 and 0.82, where the formation of the water needle occurs, DOPC:DOPS exhibits a larger flux compared to DPhPC:DPhPS. These findings suggest that the proton flux increases with the size of defects and the structure of the alkyl chains affects the proton movement in water needles. Chloride flux is also influenced by the size of defects, but there is no significant difference in the Cl^–^ flux between the DOPC:DOPS and DPhPC:DPhPS for a given degree of pore opening. Moreover, the calculated Cl^–^ flux is much lower than the H^+^ flux for a given ξ_ch_ (Figure S12). This result is in good agreement with the H^+^ to Cl^–^ permeability ratio of more than 50 that was experimentally derived from Figure 1F.

We next investigated the influence of the transmembrane potential on proton current through defects. By counting H^+^ flux across DOPC:DOPS and DPhPC:DPhPS membranes at various potentials ranging from 0 mV to 600 mV, we obtain I–V curves for the different sizes of water needles (Figure S13). First, the proton current increases with transmembrane potential, which indicates that local potentials play a crucial role in driving H^+^ ions across the water needle. Second, the change in the I–V curve is greater for larger defect sizes, which is reflected in the conductance of water needles (Figure S14). Lastly, DOPC:DOPS exhibits a higher conductance than does DPhPC:DPhPS for a given ξ_ch_. These findings suggest that for the same size of the needle, the structure of the hydrophobic core influences the proton conductance, which is in agreement with the observed proton flux results (Figure S12).

By combining the PMFs and the proton flux, we calculated the proton permeability (*P*) as a function of the reaction coordinate (ξ_ch_), which quantifies the defect connectivity and pore size:
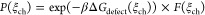
4where Δ*G*_defect_ is the free energy of defect formation, β is the inverse temperature, and *F* is the proton flux. The obtained result is shown in Figure 3B and shows that the proton permeability of DOPC:DOPS is two orders higher in magnitude than that of DPhPC:DPhPS, which exhibits a good qualitative agreement with experimental results. The proton permeation dominantly occurs in the range of ξ_ch_ ≈ 0.72–0.84 for DOPC:DOPS and ξ_ch_ ≈ 0.72–0.82 for DPhPC:DPhPS, corresponding to conformations with water needles. Structures with smaller ξ_ch_ do not conduct protons owing to a lack of water wire connectivity (Figure S12), whereas structures with larger ξ_ch_ (larger pores) are energetically unfavorable in DOPC:DOPS or DPhPC:DPhPS (Figure S10). Consequently, proton translocation does not require completely open pores but is more likely to occur through thin water needles, as illustrated in Figure 3A.

This translocation mechanism of protons is different from divalent cations like Ca^2+^ and Mg^2+^ that use the open pores for transport rather than the water needles.^26,27^ We explain this difference with the more tightly bound hydration shell of divalent ions reflected by their highly negative solvation free energies of −1250 to −2395 kJ/mol as compared to the more weakly bound hydration shell of protons, reflected by the solvation free energy of hydronium ions of only −460 kJ/mol.^70,71^ Consequently, a thin water wire may provide sufficient solvation for protons during permeation, while an open pore is required to solvate divalent ions. The fact that water needle formation requires lower free energy than pore formation may explain the higher membrane permeability of protons compared with other cations.

### 2.4 Comparing Experiments and Simulations

From the experiments, we found that there is a small amount of proton translocation that can be followed either by measuring current/conductance or by SH imaging of the interfacial water structure. The conductivity data (Figure 1F) show after 60 min, ∼5.7 × 10^–14^ moles of H^+^ passed through the DOPC:DOPS membrane while ∼0.7 × 10^–14^ moles of H^+^ moved through the same area for the DPhPC:DPhPS membrane. With SH imaging, the part of the transport process that leads to a distortion of the membrane hydrated layer can be followed, which provides information about the H^+^-lipid water complexing and how this changes in time and space. We observed spatiotemporally heterogeneous interactions (Figures 1 and 2), with spatially varying transmembrane potentials and translocation time constants varying over the membrane. We also observed different translocation behaviors for different hydrophobic cores. With the transient transmembrane potential variations as a crucial component of lipid membranes, a new perspective on proton translocation can be reached. As opposed to viewing the membrane as a static entity with fixed averaged electrostatic properties, we showed that membranes exhibit temporary local transmembrane potentials of up to ∼350 mV. Transient transmembrane potential variations lower the free energy barrier for water needle formation, however only by few kilojoule per mole for voltages up to 300 mV. If water needles form at such locations, protons can translocate, while the local transmembrane potential may also drive ion permeation of other ions (depending on the membrane structure, as we previously showed for divalent cations).^26,27^ The difference between protons and larger cations is that protons have a lower (less negative) solvation free energy as compared with divalent cations. For protons, a water needle is sufficient for permeation, while for cations transient nanopores are needed, which require more free energy to form. We previously observed such nanopore formation, and their free energy barrier/transmembrane potential is generally higher and varies for the type of ion.^26,27^ In addition, owing to the Grotthuss mechanism, the proton flux is further enhanced. The measured translocation values for H^+^ are also faster than those of divalent ions,^26,27^ which agrees with the computations. Likewise, the role of the hydrophobic core is evident from both the experiments and computations: it takes more effort to produce a water wire inside a branched membrane than inside a more loosely packed, unsaturated structure.

The fluctuations observed by SH imaging are different from the partial defects or membrane ruptures that could be expected from the application of a very high voltage. The formation of such defects is associated with a higher energy barrier, and their diameter exceeds that of the needles (see Figures S10 and S11). The fact that the fluctuations are greater for DOPC:DOPS than for DPhPC:DPhPS supports the idea that the water needles now identified may explain, at least in part, why the proton permeability often exceeds that predicted by the solubility mechanism. After all, the thickness of the two membranes is almost identical, and therefore, the “solubility diffusion model” would have predicted the proton transfer rate to be the same. The data in Figure 2 (especially 2B and 2C) clearly show that this is not the case and instead supports the “water wire” hypothesis.

Although there is a qualitative agreement between the experiment and the computation, the actual values are different. The reason could be that the measured translocation time is slow, leading to relatively small differences in the SH intensity on the time scale of the experiment. Quantitative differences between the experimental and simulated free energies can also arise from the absence of proton hopping by the Grotthuss mechanism or from the absence of electronic polarization in our simulations. The water model parametrized for bulk water properties might face limitations for modeling a thin water needle within a hydrophobic membrane core. However, considering that we recently found good agreement between simulation and experiment for the kinetics of pore formation during electroporation experiments,^72^ such force field uncertainties are probably minor.

Thus, the combination of methods together with a reinterpretation of membrane electrostatics as being a statistical/dynamic process driven by local ionic and surface chemical rearrangements rather than a constant mean-field interpretation results in a hypothesis for proton transport across lipid membranes that is similar to proton transport in ionic solutions.

## 3 Conclusions

In summary, we investigated the mechanism of proton translocation through the lipid bilayer membranes by using wide-field SH imaging, conductivity measurements, and MD simulations. A proton gradient was applied across unsaturated DOPC:DOPS and saturated branched DPhPC:DPhPS membranes. SH imaging shows potential fluctuations due to the proton membrane interaction. MD simulations report that a thin water needle is formed in a membrane defect and the potential fluctuations play a crucial role in driving H^+^ ions across the water needle. This mechanism is unique when compared to divalent cations such as Ca^2+^ and Mg^2+^ that use open pores for ion transport. The fact that water needle formation requires less free energy than pore formation may explain the higher membrane permeability of protons compared to other cations. We combined SH imaging and conductivity measurements to extract the proton permeability of DOPC:DOPS and DPhPC:DPhPS, in good qualitative agreement with simulation results. This work proposes a novel mechanism for proton translocation driven by the membrane potential via a water needle.

## Data Availability

The data that support the findings of this study are available in a public repository that issues data sets with the DOI: 10.5281/zenodo.12516968.

## Supporting Information Available

Chemicals and cleaning procedures (S1), formation of freestanding horizontal planar lipid bilayers (S2), second harmonic imaging (S3), electrical characterization (S4), SH images for DOPC:DOPS membranes (S5), conversion of SH intensity to surface potential (S6), time-averaged SH images (S7), effect of difference in ionic strength on SH and conductivity data (S8), domain analysis in the SH images (S9), calculation of surface potential arising from protonated PS lipids (S10), calculation of the average ratio of Cl^–^ to H^+^ permeability (S11), time evolution of membrane conductance and current at zero external bias (S12), setup and parameters of MD simulations (S13), free energy calculations of pore formation in DOPC:DOPS and DPhPC:DPhPS membranes (S14), free energy of water needle formation is hardly reduced by transmembrane potentials as shown by an analytic model (S15), simulation of proton permeation across a water needle and open pore (S16), proton current across a water needle vs transmembrane potential (S17), and proton conductance of water needles (S18) (PDF)
Movies of the proton translocation-induced membrane hydration for DPhPC:DPhPS (MP4)
Movies of the proton translocation-induced membrane hydration for DOPC:DOPS (MP4)
