# Supplementary material for: Dynamic Second Harmonic Imaging of Proton Translocation Through Water Needles in Lipid Membranes
Source: J Am Chem Soc. 2024 Jul 11;146(29):19818–27. doi: 10.1021/jacs.4c02810 (PMC11273352; doi:10.1021/jacs.4c02810)
Supplement: Supplementary file 1 — ja4c02810_si_001.pdf [file ja4c02810_si_001.pdf]

# Supporting Information for:

# Dynamic Second Harmonic Imaging of Proton Translocation Through Water Needles in Lipid Membranes

Seonwoo Lee[1], Chetan S. Poojari[2], Anna Maznichenko[3], David Roesel[1], Iwona Swiderska[1], Peter Pohl[3], Jochen S. Hub[2] and Sylvie Roke[1,#]

[1]Laboratory for fundamental BioPhotonics (LBP), Institute of Bio-engineering (IBI), and Institute of Materials Science (IMX), School of Engineering (STI), and Lausanne Centre for Ultrafast Science (LACUS), École Polytechnique Fédérale de Lausanne (EPFL), Lausanne CH-1015, Switzerland,

[2]Theoretical Physics and Center for Biophysics, Saarland University, Saarbrücken 66123, Germany;

[3]Institute of Biophysics, Johannes Kepler University Linz, Gruberstraße 40, Linz 4020, Austria;

*[#]Author to whom correspondence should be addressed: sylvie.roke@epfl.ch.*

**Table of Contents:**

S1. Chemicals and cleaning procedures

S2. Formation of freestanding horizontal planar lipid bilayers

S3. Second harmonic imaging

S4. Electrical characterization

S5. SH images for DOPC:DOPS membranes

S6. Conversion of SH intensity to surface potential

S7. Time averaged SH images

S8. Effect of difference in ionic strength on SH and conductivity data

S9. Domain analysis in the SH images

S10. Calculation of surface potential arising from protonated PS lipids

S11. Calculation of the average ratio of $Cl^-$ to $H^+$ permeability

S12. Time evolution of membrane conductance and current at zero external bias

S13. Setup and parameters of MD simulations

S14. Free energy calculations of pore formation in DOPC:DOPS and DPhPC:DPhPS membranes

S15. Free energy of water needle formation is hardly reduced by transmembrane potentials as shown by an analytic model

S16. Simulation of proton permeation across a water needle and open pore

S17. Proton current across a water needle vs. trans-membrane potential

S18. Proton conductance of water needles

**List of figures:**

Figure S1. The molecular structures of the lipid used in this work.

Figure S2. Formation of a lipid bilayer membrane.

Figure S3. SH images and membrane potential landscapes at different pH environments.

Figure S4. Calibration curve between SH intensity and surface potential.

Figure S5. SH images averaged over time.

Figure S6. Effect of difference in ionic strength on potential landscapes and membrane conductivity.

Figure S7. Transmembrane potential difference histograms.

Figure S8. Surface potential resulting from the protonation of PS lipids.

Figure S9. Time evolution of membrane conductance and current at zero external bias.

Figure S10. Potential of mean force of pore formation.

Figure S11. Reduction of free energy of water needles by transmembrane potentials.

Figure S12. Number of permeation events under a transmembrane potential difference from MD simulations at fixed degrees of pore nucleation and opening.

Figure S13. Proton current at varying with transmembrane potentials for different degrees of needle opening.

Figure S14. Proton conductance of water needles with different sizes.

## Experimental Section (S1-S4)

## S1. Chemicals and cleaning procedures

1,2-diphytanoyl-sn-glaycero-3-phosphocholine (DPhPC), 1,2-diphytanoyl-sn-glycero-3-phospho L-serine (DPhPS), 1,2-dioleoyl-sn-glycero-3-phosphocholine (DOPC), and 1,2-dioleoyl-sn-glycero-3-phospho-L-serine (DOPS) in powder form (>99%, Avanti Polar Lipids, Alabama, USA), hexadecane ($C_{16}H_{34}$, 99.8%, Sigma-Aldrich), hexane ($C_6H_{14}$, 99%, Sigma-Aldrich), chloroform ($CHCl_3$, 99.8%, Merck), hydrogen peroxide ($H_2O_2$, 30-32%, Reactolab SA), sulfuric acid ($H_2SO_4$, 95-97%, ISO, Merck), hydrochloric acid (HCl, 36-38%, Sigma-Aldrich), KCl (99.999%, Aros), monosodium phosphate ($NaH_2PO_4 \geq$ 99.0%, Sigma-Aldrich), disodium phosphate ($Na_2HPO_4 \geq$ 99.0%, Sigma-Aldrich) and agar ($(C_{12}H_{18}O_9)_n$, > 99%, Sigma-Aldrich) were used as received. The chemical structures of lipids are shown in Fig. S1. All aqueous solutions were made with ultra-pure water ($H_2O$, Milli-Q UF plus, Millipore, Inc.), which has an electrical resistance of 18.2 MΩcm). All aqueous solutions were filtered with 0.1 µm Millex filters. For each experiment, the coverslips were taken freshly from the packaging and were pre-cleaned by soaking them thoroughly in piranha solution (a 1:3 mixture of 30-32% $H_2O_2$ : 95-97% $H_2SO_4$) for 1 hour after which they were thoroughly rinsed with ultrapure water. For lipid membrane formation 13 mM lipid - $CHCl_3$ solutions were used, which is sufficient to make a well packed lipid monolayer on an air/water interface. Lipid mixtures were prepared by mixing the lipid-containing solutions to the desired molar ratios. To introduce a pH gradient across the membranes, 3 M HCl solutions were prepared and added to the bottom compartment of membranes using a micropipette (Eppendorf).

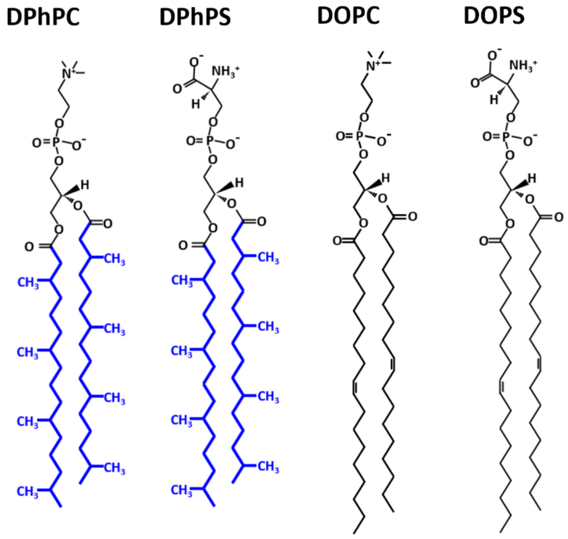


**Figure S1. The molecular structures of the lipid used in this work.** Fully saturated branched phospholipids (DPhPC and DPhPS) and mono-unsaturated unbranched phospholipids (DOPC and DOPS).

## S2. Formation of freestanding horizontal planar lipid bilayers

Freestanding horizontal planar lipid membranes were formed using the procedure of Montal and Müller[1] and modifications were applied to form horizontal instead of vertical membranes[2]. Two separate lipid monolayers were formed on two air/water interfaces that are separated by a 25-µm thick Teflon film that has a ~110-µm diameter aperture in the center. The lipid monolayers were brought in contact by rotating the Teflon film through the interfaces and a bilayer was formed in the center. The process is schematically shown in Fig. S2A. The composition of the leaflets and the aqueous solution that surround the bilayer were controllable in-situ. To facilitate membrane formation and the transition between the ~4 nm thick lipid bilayer and the 25 µm thick Teflon film, a 99.5:0.5 vol % mixture of hexane and hexadecane coating was applied to the edge of the aperture of the film. When the lipid bilayer was formed, a torus of hexadecane (the hexane evaporates) was formed close to the edge of the aperture and was visible as a Newton diffraction ring, as shown in Fig. S2B. Considering a diffusion constant of 1.8 ± 1.0 $\mu m^2$ /s for hexadecane[3] and the diameter of membranes, we waited ~$10^3$ seconds for hexadecane to move to the reservoir at the edge before the conductivity and the SH measurements.

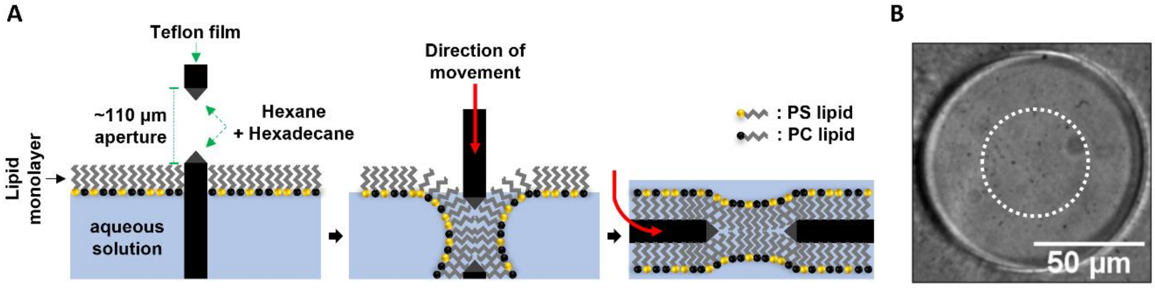


**Figure S2. Formation of a lipid bilayer membrane.** (A) Schematic illustration of the formation of a bilayer membrane. (B) A white-light image of the membrane with a Newton diffraction ring. The central part of the lipid bilayer (white circle) was used to obtain SH images and potential landscapes in this study.

## S3. Second harmonic imaging

Two beams from a Yb:KGW femtosecond laser (200 kHz repetition rate, 190 fs pulses, 1030 nm wavelength, Light Conversion Ltd) were incident under a 90° angle, each making a 45° angle with the surface normal (Fig. 1B). Each beam was loosely focused using f = 20 cm doublet lens (B coating, Thorlabs) to make a ~ 150 µm diameter excitation area. The polarization of each beam was controlled using a linear polarizer (Glan-Taylor polarizer, GT10-B, Thorlabs) and a zero-order $\lambda/2$ wave plate (WPH05M-1030, Thorlabs). The laser power for each arm was set to 160 mW. The phase-matched SH photons were collected by a 50x

objective lens (Plan Apo NIR HR Infinity-Corrected Objective, 0.65 NA; Mitutoyo), collimated with a 18 cm tube lens (MT-L; Mitutuyo) and captured by an intensified electronically amplified CCD camera (EM-ICCD, PIMax4; Princeton Instruments). A 400 mm meniscus lens was placed behind the objective lens to remove spherical aberrations induced by the coverslip. The fundamental beam and potential two-photon excited fluorescence (2PF) was blocked using a 900 nm short pass filter (FES0900; Thorlabs) and a 515-nm bandpass filter (10 nm bandwidth, FL514.5-10). Both lenses were placed in the detection path. A $\lambda/2$ wave plate and a Glan-Taylor prism were used to control the polarization of the generated SH light. All images were measured with the beams P-polarized, parallel to the plane of incidence. The acquisition time of individual frames was 1 s.

## S4. Electrical characterization

The presence of a bilayer was confirmed with white light imaging (Fig. S2B) and electrical measurement with specific capacitance, $C_m > 0.7$ µF $cm^{-2}$, specific resistance, $R_m \sim 10^7\ \Omega \cdot cm^2$ . Theoretical estimates of the specific capacitance for solvent free bilayers range from 0.75 to 0.81 µF/$cm^2$ (Ref 4). Since we measured values in this range, it is reasonable to conclude that our membranes are free of hexadecane. For membrane conductivity measurements, agar salt bridges were used to provide the stable junction potential of electrodes in contact with solutions. To prepare agar salt bridges, a mixture of 3 % agar (w/v) in a 3 M KCl solution was slowly heated up to 150 °C while stirring until the agar was completely dissolved. Polypropylene pipet tips were then immersed in the solution, and the liquid agar was drawn into the tips via a capillary force. The tips were then cooled down to 4 °C to solidify the aqueous agar. For each measurement, the freshly prepared tips were taken and back-filled with 3 M KCl solutions. Ag/AgCl electrodes that were connected to a patch-clamp amplifier (EPC-10, HEKA Electronics, Germany) were inserted into those tips, and were placed on both sides of the membrane. The current response of the lipid membrane to a given external bias was measured with a sampling frequency of 0.5 kHz. For the recording, a 4-pole Bessel filter with a frequency of 0.1 kHz was used.

## S5. SH images for DOPC:DOPS membranes

Fig. S3 shows single-frame SH images ($I_{SH}$) with the corresponding trans-membrane potential differences ($\Delta\Phi_0$) also indicated for 70:30 mol% DOPC:DOPS membranes with the top/bottom leaflet in contact with a 50 mM KCl solution with a 10 mM sodium phosphate buffer ($Na_2HPO_4$-$NaH_2PO_4$), having an identical pH for both leaflets (pH = 7.3 / pH = 7.3; image (i)) or having a

different pH value for both leaflets (pH = 4.1 / pH = 7.3; image (ii)).This data is equivalent Fig. 1D, having the same experiment but a different membrane.

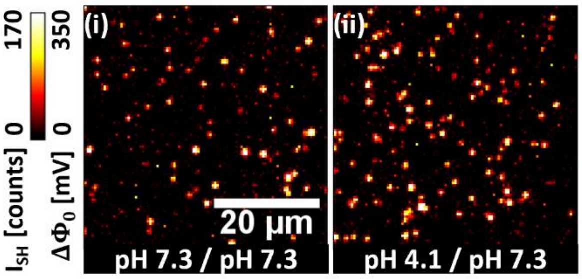


**Figure S3. SH images and membrane potential landscapes at different pH environments.** SH intensity ($I_{SH}$) and corresponding transmembrane potential difference ($\Delta\Phi_0$) images (acquisition times 1 s) of a symmetric membrane composed of a 70:30 mol% DOPC:DOPS (i) before and (ii) 10 min after the addition of $(HCl)_{aq}$ to the bottom compartment. Both sides of the bilayer are initially in contact with a pH 7.3, 50 mM KCl and 10 mM phosphate buffer solution. Upon the addition of $(HCl)_{aq}$, the pH value of the bottom compartment decreases from pH 7.3 to pH 4.1 while the pH value at the top compartment remains pH 7.3.

## S6. Conversion of SH intensity to surface potential

For a lipid membrane with two leaflets (*i* = 1 or 2), the total emitted SH intensity $I(2\omega, x, y)$ is related to the membrane surface potential on the aqueous side of each leaflet ($\Phi_{0,i}$) and expressed as[5]:

$$I(2\omega,x,y) \propto I(\omega,x,y)^2 \left|\chi_{s,1}^{(2)}(x,y) - \chi_{s,2}^{(2)}(x,y) + \chi^{(3)\prime} f_3\big(\Phi_{0,1}(x,y) - \Phi_{0,2}(x,y)\big)\right|^2 \quad (1)$$

where $\omega$ is the frequency of the fundamental beam, $x$ and $y$ are the spatial coordinates, $\chi_{s,i}^{(2)}$ (*i* = 1 or 2) are the second-order surface susceptibilities of each leaflet, $\Phi_{0,i}$ (*i* = 1 or 2) are the surface potentials of each leaflet of the membrane as seen from the aqueous side, $\chi^{(3)\prime}$ is the effective third-order susceptibility of water, and $f_3$ is an interference term determined by the geometry of the experiment. For a transmission experiment, as performed here, $f_3 = 1$. For symmetric membranes that have an identical lipid composition on each leaflet, we assume that $\chi_{s,1}^{(2)} = \chi_{s,2}^{(2)}$. Because $\chi_{s,i}^{(2)}$ does not change significantly upon the addition of ions and $\chi^{(3)\prime}$ is two orders of magnitude larger than $\chi_{s,i}^{(2)}$[5], the SH intensity observed in our images is attributed to the difference in membrane potential $\Delta\Phi_0(x,y) = \Phi_{0,1}(x,y) - \Phi_{0,2}(x,y)$ and Eq. (1) can therefore be reduced to:

$$I(2\omega,x,y) \propto I(\omega,x,y)^2 \left|\chi^{(3)\prime} \Delta\Phi_0(x,y)\right|^2. \qquad (2)$$

Based on Eq. (2), the SH intensity is converted to the transmembrane potential difference $\Delta\Phi_0(x,y) = \Phi_{0,1}(x,y) - \Phi_{0,2}(x,y)$. By recording SH images as a function of external electric bias across the membrane the intensity can be converted into a membrane potential difference. The method is described in detail in the supplementary material of Ref. 5, and results in the conversion graph of Fig. S4.

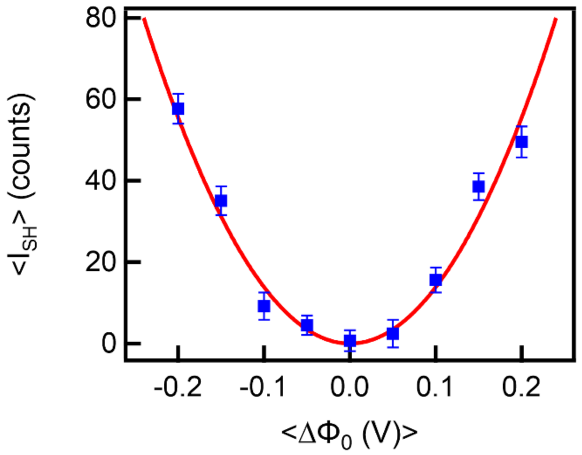


**Figure S4. Calibration curve between SH intensity and surface potential.**

## S7. Time averaged SH images

The SH images of Fig. 1 and 2 show spatiotemporal fluctuations, which arise from local symmetry breaking, induced by spatially and temporally varying charge distributions in the interfacial electric double layer regions of the top and bottom lipid membrane leaflets. Increasing the acquisition time reduces the effect of such spatial-temporal variation on the SH images, resulting in a vanishing SH intensity.

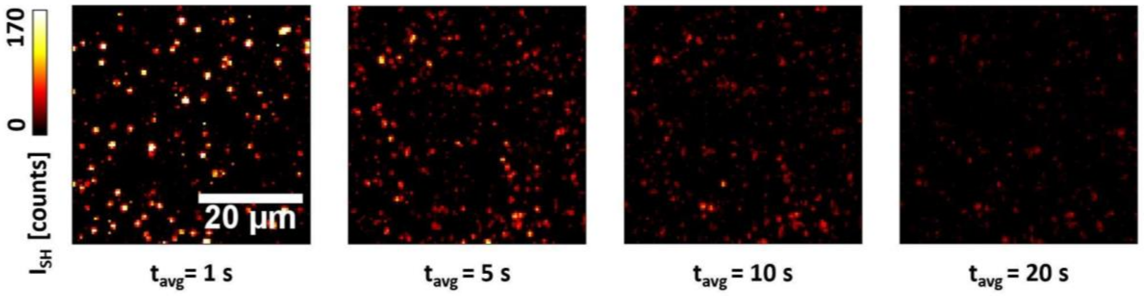


**Figure S5. SH images averaged over time.** Time averaged SH images of a symmetric membrane composed of a 70:30 mol% DPhPC:DPhPS mixture surrounded by isotropic pH 7.3, 10 mM phosphate buffer and 50 mM KCl

solution. As the average time($t_{avg}$) increases, the influence of spatial-temporal intensity variation on SH images decreases and thus SH intensity gradually vanishes.

## S8. Effect of difference in ionic strength on SH and conductivity data

Changing the pH also changes the ionic strength by ~ 1 mM. Fig. S6 shows the effect of ionic strength on the SH (Fig. S6A) and conductivity (Fig. S6B) data, by adding 1 mM KCl to the bottom compartment.

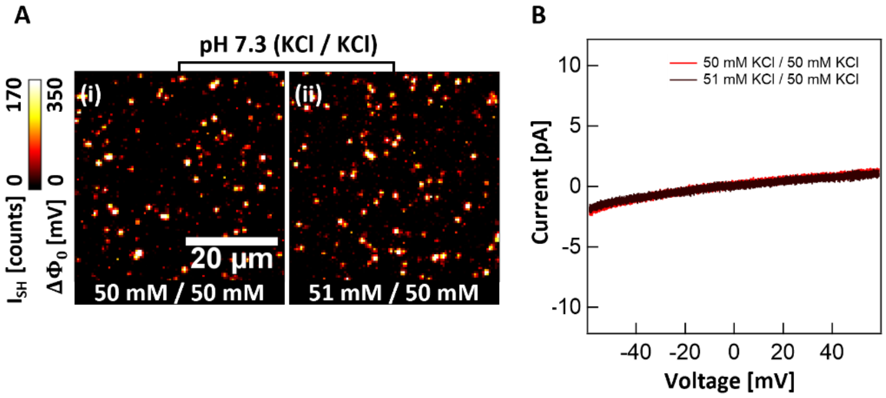


**Figure S6. Effect of difference in ionic strength on potential landscapes and membrane conductivity.** (A) SH intensity ($I_{SH}$) and corresponding transmembrane potential difference ($\Delta\Phi_0$) images (acquisition times 1 s) of a symmetric membrane composed of a 70:30 mol% DPhPC:DPhPS. (i): the top and bottom leaflet is in contact with 10 mM phosphate buffer and 50 mM KCl solution. (ii): 10 minutes after KCl concentration in the bottom compartment is changed to 51 mM. Measurements are performed under pH 7.3. (B) Membrane conductivity of 70:30 mol% DPhPC:DPhPS for different ionic concentrations as used in Fig. S6A. When the balance of ionic strength is broken, negligible changes are observed for potential landscapes and membrane conductivity. This implies the increase in membrane potential and conductivity shown in Fig. 1 and 2 is not caused by the difference in ionic strength.

## S9. Domain analysis in the SH images

For the selection of domains in our images we applied Gaussian fitting using the GDSC ImageJ plugin developed by Alex Herbert[6]. Background intensity caused by noise in the CCD camera was used as the threshold for finding the domain. The size of the domain was obtained by X and Y standard deviation from Gaussian fitting. Since two standard deviations ($2\sigma$) account for ~95% in a Gaussian distribution, the domain size was determined by an area that has 2x the standard deviation in both X and Y axis. The average SH intensity of each domain was then obtained. The following parameters were used for the fitting: smoothing = 0, box size=2, background = 550, min height = 0, fraction above background = 0, min height = 0, top n =0, neighbor check border = 2, fit function = [Free circular], fit criteria = [Least squared error],

max iterations = 30, significant digits = 1, coord delta = 0.01, single region size = 5, and initial stdev = 0. After the SH domains were selected, the pixel-averaged SH intensity/domain was converted to the transmembrane potential/domain ($\Delta\Phi_{domain}$). Finally, the domains were organized in terms of the occurrence of certain membrane potential differences, producing a histogram. Fig. 1E shows the result for the DPhPS:DPhPC membrane and Fig. S7 shows the results for the DOPS:DOPC membrane.

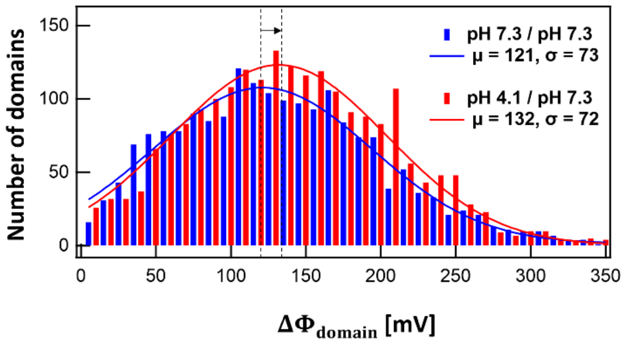


**Figure S7. Transmembrane potential difference histograms.** The number of domains with a given membrane potential of the domain ($\Delta\Phi_{domain}$) observed in 20 x 1s SH images of the symmetric membrane composed of 70:30 mol% DOPC:DOPS in contact with a 50 mM KCl and phosphate buffer solution before (pH = 7.3) and 10 minutes after (pH = 4.1) addition of HCl to the bottom compartment. Data points are fitted with Gaussian distribution, and the mean (μ) and standard deviation (σ) are displayed. The shift of the entire distribution is indicated by the arrow.

## S10. Calculation of surface potential arising from protonated PS lipids

To understand the electrostatic behaviour of a lipid bilayer membrane having a single charged leaflet in contact with a neutral one we use the Gouy-chapman-Stern (GCS) model. The GCS model[7] relates the surface potential of a charged planar leaflet ($\Phi_0$) in an aqueous medium to the surface charge density ($\sigma_0$), and the ionic strength in the solution ($c$) for symmetric 1:1 electrolytes:

$$\Phi_0 = \frac{\sigma_0 d_s}{\varepsilon_0 \varepsilon_r{}'} + \frac{2k_B T}{e} sinh^{-1}\left(\frac{\sigma_0}{\sqrt{8000 k_B T N_A c \varepsilon_0 \varepsilon_r}}\right),$$

with $d_s$, $\varepsilon_0$, $\varepsilon_r{}'$, $\varepsilon_r$, $k_B$, $T$, $e$, $N_A$, the Stern layer thickness, the permittivity of vacuum, the dielectric constant in the Stern layer, the dielectric constant of the bulk medium (water), the Boltzmann constant, temperature, elementary charge and Avogadro's number. Taking the dielectric constant in the Stern layer to be $\varepsilon_r{}'$ = 43 from Ref 8. For the other constants we used the values: $\varepsilon_0$ = 8.85·10$^{-12}$ C V$^{-1}$m$^{-1}$, $\varepsilon_r$ = 78, $k_B$ = 1.38·10$^{-23}$ J K$^{-1}$, $T$ = 295 K, $e$ = 1.6·10$^{-19}$ C, and $N_A$ = 6.02·10$^{23}$ mol$^{-1}$. Fig. S8 shows the surface potential of a surface in contact with an

aqueous solution having an ionic strength of 0.06 M, computed using the GCS model as a function of surface charge density (bottom axis). The surface charge density is also converted to the area per charge (top axis), assuming 70 Å$^2$ per one charge, which is an estimation for the cross-sectional area of one PS lipid molecule. Note that the charge density at the lipid headgroup plane is used for the conversion instead of that at the Stern plane. The graphs are computed for different thicknesses of the Stern layer $d_s$, between 0 and 0.9 nm, with 0.3 nm intervals. The surface potential increases for a given $\sigma_0$ as the thickness of Stern layer increases. In absence of a Stern layer a maximum of $\Phi_0$ = -141 mV is computed. To achieve values higher than this, a Stern or condensed layer of ions needs to be present. Also, the SH images suggest the presence of a fluctuating non-uniform charge distribution, which confirms previous estimations[5,9].

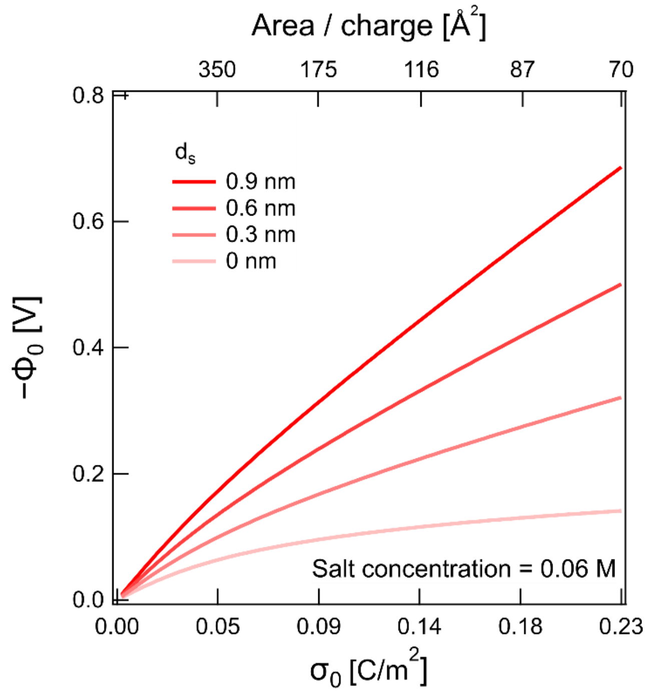


**Figure S8. Surface potential resulting from the protonation of PS lipids.** Surface potential ($-\Phi_0$) increases for a given surface charge density ($\sigma_0$) of PS lipids, related to area per charge, as the thickness of the stern layer ($d_s$) increases.

## S11. Calculation of the average ratio of $Cl^-$ to $H^+$ permeability

To calculate the average ratio of $Cl^-$ to $H^+$ permeability, we applied the reversal potentials of 15 mV for DOPC:DOPS and 2 mV for DPhPC:DPhPS to eq. 1 in the manuscript. The eq.1 can be converted to the following equation:

$$(P_{Cl^-}/P_{H^+})= \frac{\exp\left\{V_m/(\frac{RT}{F})\right\} c^t_{H^+} - c^b_{H^+}}{-\exp\left\{V_m/(\frac{RT}{F})\right\} c^b_{Cl^-} + c^t_{Cl^-}}$$

where $R$ is the ideal gas constant, $T$ is the temperature, $F$ is the Faraday's constant, and $c^b_{ion}$ and $c^t_{ion}$ are the ion concentrations of the bottom and top compartments, respectively. For the constants, we used the values: $R = 8.31\ \mathrm{J \cdot mol^{-1} K^{-1}}$, $T = 293$ K, $F = 9.648 \cdot 10^4 \mathrm{C \cdot mol^{-1}}$, $c^t_{H^+}$=$10^{-7.3}$M, $c^b_{H^+} = 10^{-4.1}$M, $c^b_{Cl^-}$= 0.051 M, $c^t_{Cl^-}$= 0.05 M. For the 2 mV case, $P_{Cl^-}/P_{H^+}$= 0.015, and for the 15 mV case, $P_{Cl^-}/P_{H^+}$= 0.002. These findings suggest that the average permeation rate of protons is significantly higher than that of chlorides.

## S12. Time evolution of membrane conductance and current at zero external bias

To quantify proton conductivity (Fig. S9A), and the current (Fig. S9B) through the membrane, the slope of the graphs in Fig. 1F was used together with the membrane area ($\pi(55)^2$= 9503 $\mu m^2$). Before adding $(HCl)_{aq}$, at an identical pH for both leaflets (pH = 7.3), DOPC:DOPS has a higher conductivity ($2.4 \cdot 10^{-7}$ S/cm$^{-2}$) than DPhPC:DPhPS ($1.5 \cdot 10^{-7}$ S/cm$^{-2}$). After adding 1 mM $(HCl)_{aq}$, the conductance of both membranes increases, with DOPC:DOPS exhibiting a value that is roughly twice as high as that of DPhPC:DPhPS ($11.2 \cdot 10^{-7}$ S/cm$^{-2}$ vs. $5.5 \cdot 10^{-7}$ S/cm$^{-2}$, respectively). The current shows a similar difference.

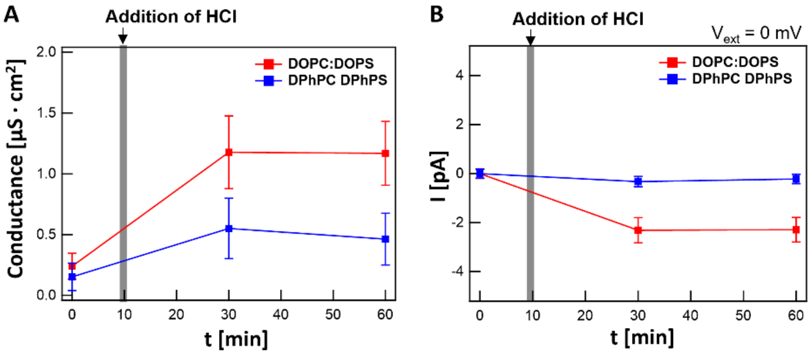


**Figure S9. Time evolution of membrane conductance and current at zero external bias.** Using current-voltage (I-V) curves shown in Fig. 1F, the time evolution of (A) membrane conductance and (B) current at zero external bias for symmetric membranes composed of 70:30 mol% DOPC:DOPS (red) and 70:30 mol% DPhPC:DPhPS (blue) before and after addition of 1 mM $(HCl)_{aq}$ to the bottom compartment.

## S13. Setup and parameters of MD simulations

DOPC:DOPS (70:30 mol%) and DPhPC:DPhPS (70:30 mol%) membranes were prepared with the MemGen[10] web server with 162 lipids and 40 Charmm-modified TIP3P water molecules per lipid[11]. As used in our previous studies on ion flux[12,13], the simulation systems were described with the Charmm36 lipid force field with electronic continuum correction (Charmm36-ECC), a Charmm36 variant with improved lipid–ion interactions[14-16]. Electrostatic interactions were described with the particle-mesh Ewald method[17-18]. Lennard-Jones interactions were truncated at 1.2 nm, while the force was gradually switched off between 1 and 1.2 nm. The simulation systems were first energy minimized using steepest descent algorithm, followed by equilibration with temperature controlled at 310 K using velocity rescaling[19], thereby coupling membrane and solvent to separate heat baths (τ = 1 ps). The pressure was kept at 1 bar using the semi-isotropic Parrinello-Rahman barostat[20]. The geometry of water molecules was constrained with the SETTLE algorithm[21]. Other bonds involving hydrogen atoms were constrained with the LINCS algorithm[22]. The membranes were equilibrated for 200 ns with an integration time step of 2 fs using GROMACS simulation software, version 2021[23].

## S14. Free energy calculations of pore formation in DOPC:DOPS and DPhPC: DPhPS membranes

Potentials of mean force (PMFs) of pore formation were computed along the chain coordinate $\xi_{ch}$, a reaction coordinate that quantifies the degree of connectivity of a polar defect over the lipid membrane[24,25]. In brief, $\xi_{ch}$, is defined with the help of a cylinder that spans the lipid membrane. The cylinder is decomposed into $N_s$ slices. $\xi_{ch}$ is defined by the fraction of slices that are filled by water oxygen atoms. By pulling the simulation system along $\xi_{ch}$, the slices are filled one-by-one, thereby forming a continuous polar defect over the membrane. The lateral position of the cylinder is dynamically defined, thereby allowing the cylinder to follow the defect as the defect travels laterally in the membrane plane. Harmonic restraints along $\xi_{ch}$ as required or umbrella sampling simulations have been implemented into an in-house modification of GROMACS 2021, which is freely available together with documentation and tutorials at https://gitlab.com/cbjh/gromacs-chain-coordinate. Technical details on $\xi_{ch}$ have been described in Refs. 25-27.

Starting from an equilibrated membrane, a pore was introduced at 0 mV and 300 mV using constant-velocity pulling from $\xi_{ch} = 0.1$ to $\xi_{ch} = 1$ over 100 ns, using a force constant of 3000 kJ/mol along $\xi_{ch}$. The transmembrane potential ($\Delta\Phi_0$) was implemented using an external electric field $E_z$ along the $z$-direction (membrane normal)[28]. Here, $E_z$ was chosen such that

$E_zL_z$ equals the transmembrane potential, where $L_z$ is the box dimension in $z$ direction. We showed recently that using such external electric fields yields identical PMFs as compared to simulation that impose a transmembrane potential using charge imbalance[29]. The coordinate $\xi_{ch}$ was defined with a cylinder with radius $R_{cyl}$ = 0.9 nm, decomposed into slices with a thickness of $d_s$ = 0.1 nm. Upon the addition of the first polar atom, a slice was considered as being filled by 75%. The number of slices $N_s$, and thereby the length of the cylinder $N_sd_s$, was chosen such that $\xi_{ch} \approx 0.1$ for the flat unperturbed membrane, implying that approximately 10 % of the slices are filled by polar atoms in the unperturbed membrane. Critically, the parameter $R_{cyl}$ used to define the $\xi_{ch}$ does not impose a certain radius of the defect but merely serves to ensure the locality of the defect in the membrane plane. Instead, the structure of the water needle or of the open pore is –for a given $\xi_{ch}$– controlled by the force field (together with simulation parameters such as temperature, constraints etc).

The PMFs were computed using umbrella sampling with 27 umbrella windows. Starting frames for umbrella sampling were taken from the constant-velocity pulling simulation. Since the conformational sampling is more challenging at larger $\xi_{ch}$ values compared to smaller $\xi_{ch}$ values, we used the following non-equidistant spacing for the reference positions of the umbrella windows: 0.065 through 0.64 in steps of 0.08, and 0.64 through 1.0 in steps of 0.02. For reference positions smaller or larger than 0.7 we used force constants of 5000 or 10000 kJ/mol, respectively. Each window was simulated for 100 ns. The first 20 ns were omitted for equilibration, and the PMFs were computed with the weighted histogram analysis method, as implemented in the gmx wham module of GROMACS[30,31]. Statistical errors were estimated using 50 rounds of bootstrapping complete histograms.

We recently found excellent agreement between (i) free energies of pore formation across a membrane of DPhPC obtained with our reaction coordinate together with the Charmm36 lipid force field[32] with (ii) experimental kinetic data of electroporation[33]. This agreement suggests that the free energies of pore or water needle formation reported reasonably agree with experimental conditions.

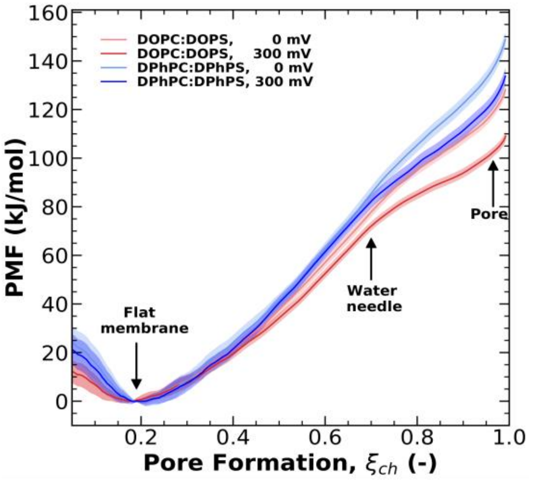


**Figure S10. Potential of mean force of pore formation**. PMFs of water needle and pore formation through DOPC:DOPS and DPhPC:DPhPS membranes at different membrane potentials ($\Delta\Phi_0$). The PMF is plotted as function of the reaction coordinate ($\xi_{ch}$) that quantifies the transition from a flat membrane, over the water needle, and up to an open pore are indicated by the arrows.

## S15. Free energy of water needle formation is hardly reduced by transmembrane potentials as shown by an analytic model

To rationalize the small effect of the transmembrane potentials up to 300 mV on the formation of a water needle, we model needles as membrane-spanning cylinders of radius *r*[32,34]. The formation of the needle changes the capacitance of the membrane by:

$$\Delta C = \pi r^2 (\varepsilon_w - \varepsilon_m)/d,$$

where $\varepsilon_w$ and $\varepsilon_m$ denote the dielectric permitivities of water and of the membrane core, and *d* is the membrane thickness. Then, the free energy of the needle is stabilized by the potential U by $\Delta E_{pot} = -\frac{1}{2}\Delta C U^2$. We assume relative permitivities of 80 and 2 for water and for the membrane core, respectively. Plotting $\Delta E_{pot}$ versus potential *U* for needles with radii of 1 Å, 2 Å, or 3 Å reveals that transmembrane potentials of few hundred millivolts stabilize the needle by only few kilojoules per mole, in good agreement with the PMFs of pore formation obtained with MD simulations.

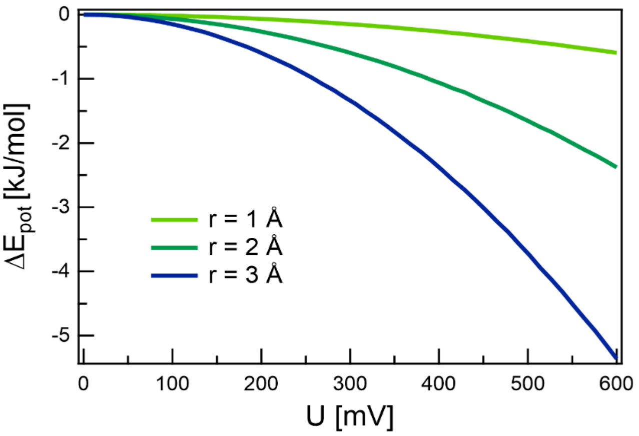


**Figure S11. Reduction of free energy of water needles by transmembrane potentials.** The change in the free energy of needle formation with radii (*r*) of 1, 2, and 3 Å for potentials (*U*) between 0 and 600 mV. Thus, for potentials up to 300 mV observed by SH data, water needle formation is stabilised by only a few kilojoules per mole, in agreement with the PMFs obtained from MD simulations (Fig. S10).

## S16. Simulation of proton permeation across a water needle and open pore

Systems for the simulation of $H^+$ permeation across a defect were set up as described in S13. Water molecules were replaced with $H_3O^+$ to obtain $H_3O^+$ concentrations of 600 mM and the system charge was neutralized by adding $Cl^-$ ions. $Cl^-$ and $H_3O^+$ ion parameters were taken from Refs. 35-37. We deliberately used large $H_3O^+$ ion concentrations to improve the statistics of permeation events. After 200 ns equilibration, a pore was induced at a transmembrane potential of 300 mV by pulling along the $\xi_{ch}$ from 0.1 to 1 over 100 ns using a force constant of 3000 kJ/mol. Then, the system was restrained at various $\xi_{ch}$-positions with a harmonic restraint along $\xi_{ch}$ with a force constant of 10000 kJ/mol, and then simulated for 500 ns. The flux at various $\xi_{ch}$-positions was obtained by counting the number of $H_3O^+$ and $Cl^-$ permeation events at a constant potential of 300mV (Fig. S12).

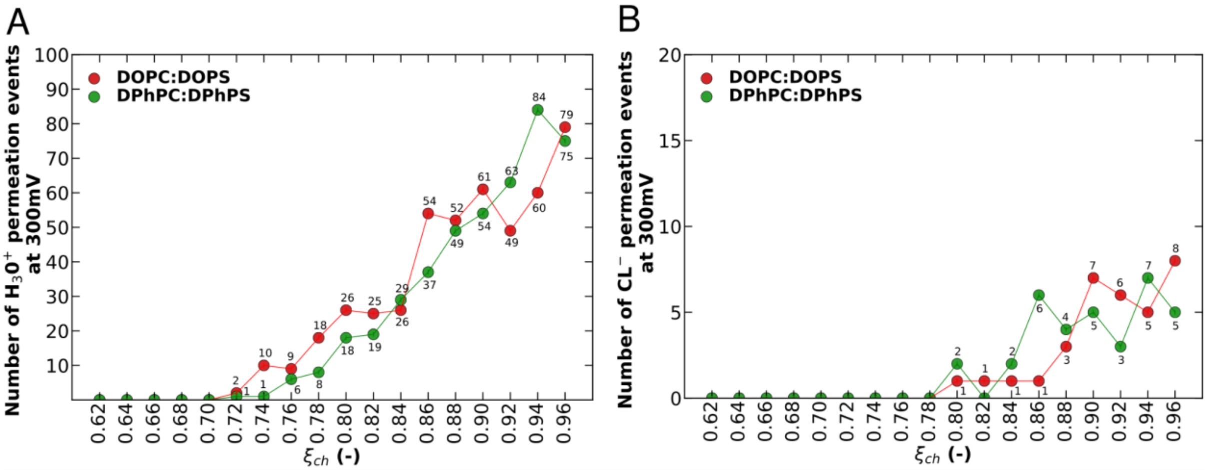

**Figure S12. Number of permeation events under a transmembrane potential difference from MD simulations at fixed degrees of pore nucleation and opening.** Cumulative number of (A) $H_3O^+$ and (B) $Cl^-$ permeation events over 500 ns for membranes at 300 mV at different $\xi_{ch}$.

## S17. Proton current across a water needle vs. trans-membrane potential

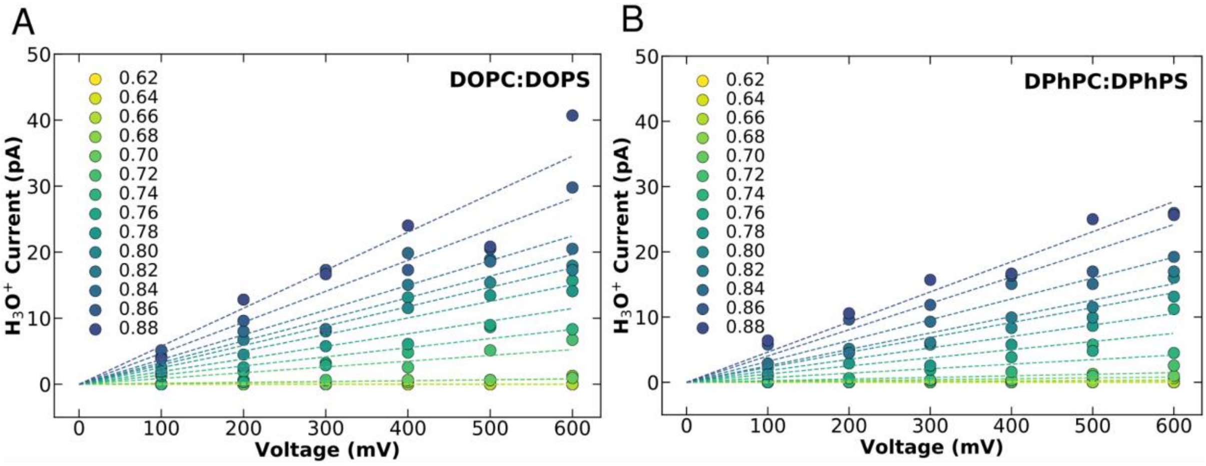


**Figure S13. Proton current at varying transmembrane potentials for different degrees of needle opening.** Proton current through (A) DOPC:DOPS and (B) DPhPC:DPhPS membranes in a range of transmembrane potentials from 0 mV to 600 mV, at different degrees of pore opening as given by $\xi_{ch}$ values ranging from 0.62 to 0.88. The simulations with various transmembrane potentials (100 mV - 600 mV) were carried out as described in S16.

## S18. Proton conductance of water needles

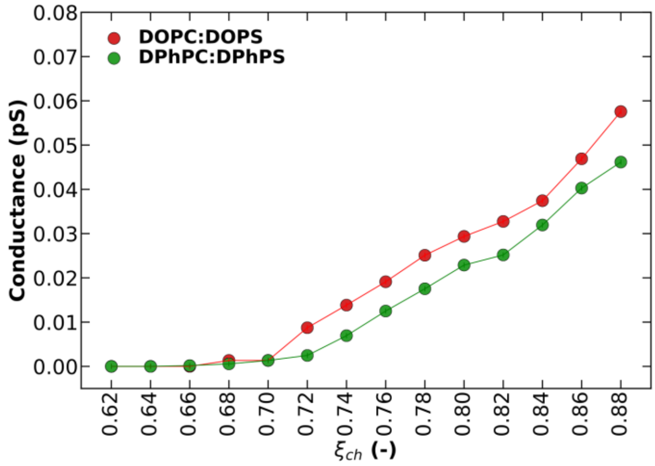


**Figure S14. Proton conductance of water needles with different sizes.** Proton conductance in DOPC:DOPS and DPhPC:DPhPS symmetric membranes at different $\xi_{ch}$ values ranging from 0.62 to 0.88. The slope of the graphs in Fig. S13 corresponds to the conductance for a given $\xi_{ch}$ value shown here.

## References


(1) Montal, M.; Mueller, P. Formation of Bimolecular Membranes from Lipid Monolayers and a Study of Their Electrical Properties. *Proc. Natl. Acad. Sci.* **1972**, 69 (12), 3561–3566.
(2) Horner, A.; Akimov, S. A.; Pohl, P. Long and Short Lipid Molecules Experience the Same Interleaflet Drag in Lipid Bilayers. *Phys. Rev. Lett.* **2013**, 110 (26), 268101.
(3) Tarun, O. B.; Eremchev, M. Y.; Roke, S. Interaction of Oil and Lipids in Freestanding Lipid Bilayer Membranes Studied with Label-Free High-Throughput Wide-Field Second-Harmonic Microscopy. *Langmuir* **2018,** 34(38), 11305-11310.
(4) White, S. H. Formation of solvent-free black lipid bilayer membranes from glyceryl monooleate dispersed in squalene. *Biophys. J.* **1978,** 23(3), 337–347.
(5) Tarun, O. B.; Hannesschläger, C.; Pohl, P.; Roke, S. Label-Free and Charge-Sensitive Dynamic Imaging of Lipid Membrane Hydration on Millisecond Time Scales. *Proc. Natl. Acad. Sci.* **2018**, 115 (16), 4081–4086.
(6) A. Herbert, A. Genome Damage and Stability Center (GDSC) Plugin (http://www.sussex.ac.uk/gdsc/intranet/microscopy/imagej/gdsc_plugins).
(7) Lütgebaucks, C.; Gonella, G.; Roke, S. Optical Label-Free and Model-Free Probe of the Surface Potential of Nanoscale and Microscopic Objects in Aqueous Solution. *Phys. Rev. B* **2016**, 94 (19), 195410.
(8) Brown, M. A.; Goel, A.; Abbas, Z. Effect of Electrolyte Concentration on the Stern Layer Thickness at a Charged Interface. *Angew. Chem.* **2016**, 128 (11), 3854–3858.
(9) Tarun, O. B.; Okur, H. I.; Rangamani, P.; Roke, S. Transient domains of ordered water induced by divalent ions lead to lipid membrane curvature fluctuations. *Commun. Chem.* **2020** 3 (1), 17.
(10) Knight, C. J.; Hub, J. S. MemGen: A General Web Server for the Setup of Lipid Membrane Simulation Systems. *Bioinformatics* **2015**, 31 (17), 2897–2899.
(11) Jorgensen, W. L.; Chandrasekhar, J.; Madura, J. D.; Impey, R. W.; Klein, M. L. Comparison of Simple Potential Functions for Simulating Liquid Water. *J. Chem. Phys.* **1983**, 79 (2), 926–935.
(12) Roesel, D.; Eremchev, M.; Poojari, C. S.; Hub, J. S.; Roke, S. Ion-Induced Transient Potential Fluctuations Facilitate Pore Formation and Cation Transport through Lipid Membranes. *J. Am. Chem. Soc.* **2022**, 144 (51), 23352–23357.
(13) Eremchev, M.; Roesel, D.; Poojari, C. S.; Roux, A.; Hub, J. S.; Roke, S. Passive Transport of Ca2+ Ions through Lipid Bilayers Imaged by Widefield Second Harmonic Microscopy. *Biophys. J.* **2023**, 122 (4), 624–631.
(14) Pastor, R. W.; MacKerell, A. D. Development of the CHARMM Force Field for Lipids. *J. Phys. Chem. Lett.* **2011**, 2 (13), 1526–1532.
(15) Melcr, J.; Martinez-Seara, H.; Nencini, R.; Kolafa, J.; Jungwirth, P.; Ollila, O. H. S. Accurate Binding of Sodium and Calcium to a POPC Bilayer by Effective Inclusion of Electronic Polarization. *J. Phys. Chem. B* **2018**, 122 (16), 4546–4557.
(16) Nencini, R.; Tempra, C.; Biriukov, D.; Polak, J.; Ondo, D.; Heyda, J.; Ollila, O. H. S.; Javanainen, M.; Martinez-Seara, H. Prosecco: polarization reintroduced by optimal scaling of electronic continuum correction origin in MD simulations. Available at: https://gitlab.com/sparkly/prosecco/prosECCo75. Accessed Oct 19, **2022**. (accessed 2022-10-19).
(17) Essmann, U.; Perera, L.; Berkowitz, M. L.; Darden, T.; Lee, H.; Pedersen, L. G. A Smooth Particle Mesh Ewald Method. *J. Chem. Phys.* **1995**, 103 (19), 8577–8593.
(18) Darden, T.; York, D.; Pedersen, L. Particle Mesh Ewald: An N·log(N) Method for Ewald Sums in Large Systems. *J. Chem. Phys.* **1993**, 98 (12), 10089–10092.
(19) Bussi, G.; Donadio, D.; Parrinello, M. Canonical Sampling through Velocity Rescaling. *J. Chem. Phys.* **2007**, 126 (1), 014101.
(20) Parrinello, M.; Rahman, A. Polymorphic Transitions in Single Crystals: A New Molecular Dynamics Method. *J. Appl. Phys.* **1981**, 52 (12), 7182–7190.
(21) Miyamoto, S.; Kollman, P. A. Settle: An Analytical Version of the SHAKE and RATTLE

Algorithm for Rigid Water Models. *J. Comput. Chem.* **1992**, 13 (8), 952–962.
(22) Hess, B. P-LINCS: A Parallel Linear Constraint Solver for Molecular Simulation. *J. Chem. Theory Comput.* **2008**, 4 (1), 116–122.
(23) Abraham, M. J.; Murtola, T.; Schulz, R.; Páll, S.; Smith, J. C.; Hess, B.; Lindahl, E. GROMACS: High Performance Molecular Simulations through Multi-Level Parallelism from Laptops to Supercomputers. SoftwareX **2015**, 1–2, 19–25.
(24) Hub, N. A., J. S. Free-Energy Calculations of Pore Formation in Lipid Membranes. In Biomembrane Simulations; CRC Press, **2019**.
(25) Hub, J. S.; Awasthi, N. Probing a Continuous Polar Defect: A Reaction Coordinate for Pore Formation in Lipid Membranes. *J. Chem. Theory Comput.* **2017**, 13 (5), 2352–2366.
(26) Awasthi, N.; Hub, J. S. Simulations of Pore Formation in Lipid Membranes: Reaction Coordinates, Convergence, Hysteresis, and Finite-Size Effects. *J. Chem. Theory Comput.* **2016**, 12 (7), 3261–3269.
(27) Ting, C. L.; Awasthi, N.; Müller, M.; Hub, J. S. Metastable Prepores in Tension-Free Lipid Bilayers. *Phys. Rev. Lett.* **2018**, 120 (12), 128103.
(28) Gumbart, J.; Khalili-Araghi, F.; Sotomayor, M.; Roux, B. Constant Electric Field Simulations of the Membrane Potential Illustrated with Simple Systems. *Biochim. Biophys. Acta BBA - Biomembr.* **2012**, 1818 (2), 294–302.
(29) Kasparyan, G.; Hub, J. S. Equivalence of Charge Imbalance and External Electric Fields during Free Energy Calculations of Membrane Electroporation. *J. Chem. Theory Comput.* **2023**, 19 (9), 2676–2683.
(30) Hub, J. S.; de Groot, B. L.; van der Spoel, D. G_wham—A Free Weighted Histogram Analysis Implementation Including Robust Error and Autocorrelation Estimates. *J. Chem. Theory Comput.* **2010**, 6 (12), 3713–3720.
(31) Kumar, S.; Rosenberg, J. M.; Bouzida, D.; Swendsen, R. H.; Kollman, P. A. THE Weighted Histogram Analysis Method for Free-Energy Calculations on Biomolecules. I. The Method. *J. Comput. Chem.* **1992**, 13 (8), 1011–1021.
(32) Kasparyan, G.; Hub, J. S. Molecular Simulations Reveal the Free Energy Landscape and Transition State of Membrane Electroporation. *bioRxiv* February 3, **2023**, p 2023.01.31.526495.
(33) Melikov, K. C.; Frolov, V. A.; Shcherbakov, A.; Samsonov, A. V.; Chizmadzhev; Y. A.; Chernomordik L. V. Voltage-induced nonconductive pre-pores and metastable single pores in unmodified planar lipid bilayer. *Biophys. J.* **2001**, 80(4), 1829-1836.
(34) Kotnik, T.; Rems, L.; Tarek, M.; Miklavčič, D. Membrane Electroporation and Electropermeabilization: Mechanisms and Models. Annu. Rev. Biophys. **2019**, 48 (1), 63–91.
(35) Bonthuis, D. J.; Mamatkulov, S. I.; Netz, R. R. Optimization of Classical Nonpolarizable Force Fields for OH− and H3O+. *J. Chem. Phys.* **2016**, 144 (10), 104503.
(36) Chew, A. K.; Van Lehn, R. C. Quantifying the Stability of the Hydronium Ion in Organic Solvents With Molecular Dynamics Simulations. *Front. Chem.* **2019**, 7.
(37) Kohagen, M.; Mason, P. E.; Jungwirth, P. Accurate Description of Calcium Solvation in Concentrated Aqueous Solutions. *J. Phys. Chem. B* **2014**, 118 (28), 7902–7909.
